# Systematic analysis of the burden of chronic kidney disease due to type 2 diabetes attributable to dietary risks based on the global burden of disease study 2021

**DOI:** 10.3389/fnut.2025.1572610

**Published:** 2025-05-21

**Authors:** Yanli Hou, Lingzhi Qin, Xuting Jin, Jiajia Ren, Jiamei Li, Xiaoling Zhang, Jingjing Zhang, Ruohan Li, Ya Gao, Xiaochuang Wang, Gang Wang

**Affiliations:** ^1^Department of Critical Care Medicine, The Second Affiliated Hospital of Xi’an Jiaotong University, Xi’an, Shaanxi, China; ^2^Key Laboratory of Surgical Critical Care and Life Support (Xi’an Jiaotong University), Ministry of Education, Xi’an, Shaanxi, China

**Keywords:** global burden of disease, chronic kidney disease due to type 2 diabetes, dietary risks, mortality, disability-adjusted life years

## Abstract

**Background:**

Dietary factors play a crucial role in the development of chronic kidney disease due to diabetes mellitus type 2 (T2D-related CKD). However, comprehensive data on the global burden of T2D-related CKD attributable to dietary risks remain limited.

**Methods:**

This study conducted a secondary analysis of the Global Burden of Diseases, Injuries, and Risk Factors Study 2021. Mortality and disability-adjusted life years (DALYs) of T2D-related CKD attributable to dietary risks, stratified by sex, age and sociodemographic index (SDI) quantiles, were analyzed in global, 21 regions, and 204 countries and territories from 1990 to 2021.

**Results:**

In 2021, 79,990 (95% confidence interval [*CI*]: 32,730–128,880) death and 1,999,210 (95% *CI*: 856,190–3,167,220) DALYs of T2D-related CKD were attributable to dietary risk factors, approximately 2.5 times as many as those in 1990. The age-standardized mortality rate (ASMR) and age-standardized DALY rate (ASDR) grew with an estimated annual percentage change (EAPC) of 0.76% (95%*CI*: 0.69–0.83%) and 0.47% (95%*CI*: 0.41–0.53%). The low SDI regions experienced the highest burden of T2D-related CKD attributable to dietary risks, with ASMR of 1.16 (95% *CI*: 0.44–2.02) per 100,000 persons and the ASDR of 27.41 (95% *CI*: 11.32–46.78) per 100,000 persons. Males and the elderly over 70 years demonstrated a higher burden of T2D-related CKD influenced by dietary risks. Diet low in fruits, whole grains, and vegetables, as well as diet high in red and processed meat serve as the main dietary risks contributed to the burden of T2D-related CKD.

**Conclusion:**

Dietary factors play a significant role in the development of T2D-related CKD. Further strategies should focus on men, the elderly, low-SDI regions and specific dietary components to mitigate dietary risks associated with T2D-related CKD.

## 1 Introduction

In recent decades, Type 2 diabetes mellitus (T2DM) has emerged as the tenth leading cause of death globally, with a significant enhance in disability burden since 1990 (1). The disease burden in individuals with T2DM predominantly stems from diabetes-induced organ dysfunction and failure, including kidneys, eyes, and nerves (2), and within the spectrum of complications, chronic kidney disease due to type 2 diabetes (T2D-related CKD) is identified as the primary driver of elevated death rates and disability-adjusted life years (DALYs) among T2DM patients with diabetic comorbidities (3, 4). From 1990 to 2021, there was a significantly increase on T2D-related CKD cases, moving from the 49th to the 25th leading cause of death and from the 103rd to the 55th leading cause of DALYs (5). The burden of T2D-related CKD approximately doubled by 2021 compared to 1990, accounting for 477,000 deaths and 11.3 million DALYs (6). Although the overall burden of T2D-related CKD continued to rise, the trend across different countries and regions exhibited notable variations, which have suggested gaps in the current status of CKD prevention and management capabilities over the world (7). Therefore, accurately assessing global trends and risk factors in T2D-related CKD is crucially essential. This will facilitate evidence-based resource allocation for effective prevention and intervention measures worldwide (8, 9).

Monitoring and managing modifiable risk factors, such as unhealthy lifestyle habits, can be a cost-effective approach for the prevention and intervention of T2D-related CKD (10). Dietary risks, defined as the consumption of food and nutrient, are recognized as potential risk factors for T2D-related CKD and offer opportunities for targeted intervention (3). Previous researches indicated that high sodium intake remained an important dietary risk factor for the global CKD burden, particularly in males, the elderly, and the population in the middle sociodemographic index (SDI) regions (11–13), which have contributed to the ongoing global increase in the burden of CKD. Targeting dietary factors in prevention and intervention efforts could effectively reduce the burden of CKD (14). Furthermore, substantial evidence exists regarding the impact of high red and processed meat consumption and inadequate whole grain, fruit, and vegetable intake on T2D-related CKD progression (15–20). However, it lacks comprehensive researches to definitively establish the relationship between dietary risks and T2D-related CKD. Moreover, focusing solely on specific dietary risks does not adequately capture the global spatiotemporal patterns of dietary influences on the T2D-related CKD burden (21–23). Further investigation is necessary to completely evaluate the impact of dietary risk factors on the T2D-related CKD burden.

In this context, we conducted a comprehensive analysis of the impact of dietary risks on T2D-related CKD by considering seven dietary risk factors across 21 Global Burden of Disease (GBD) regions, 5 SDI regions, and 204 countries and territories. The objective was aimed to present a detailed report of the global landscape of T2D-related CKD attributable to seven dietary risk factors, enhancing a deeper understanding of the role of dietary risks in terms of mortality and disability.

## 2 Methods

### 2.1 Data source and measures of burden

Data on the burden of T2D-related CKD attributable to dietary risks utilized in this study were obtained from the Global Burden of Diseases, Injuries, and Risk Factors Study 2021 (GBD 2021) (24). The GBD 2021 study provides a comprehensive and up-to-date estimation of the epidemiology of diseases, injuries and risk factors across different sexes and age groups. It covers 204 countries and territories, including 371 diseases and injuries across 21 regions, and leverages 328,938 data sources from 1990 to 2021. Details about the study design and methods of GBD studies have been extensively described in existing GBD literature (1, 11). Data from the GBD studies are publicly accessible and can be analyzed using the Global Health Data Exchange query tool.

### 2.2 T2D-related CKD

According to the GBD Study 2021 guidelines, diabetes is defined as a fasting blood glucose concentration of ≥126 mg/dL (7 mmol/L) or diabetes treatment reported (25). T2D-related CKD is defined as chronic kidney disease resulting from T2DM, characterized by a progressive decline in kidney function over a period of 3 months or more. It is primarily identified by an urinary albumin-to-creatinine ratio of ≥30 mg/g and/or an estimated glomerular filtration rate (eGFR) of <60 mL/min per 1.73 m^2^ (26).

### 2.3 Dietary risk factors

In GBD 2021, risk factors were categorized as behavioral, environmental and occupational, and metabolic. Dietary risks belong to behavioral risks. For the burden of T2D-related CKD, data on seven specific dietary risk factors were gathered: a diet low in fruits, vegetables, whole grains, and high in red meat, processed meat, sodium, and sugar-sweetened beverages. To address specific biases in data sources for estimating nutrient intake, GBD 2021 employed a network meta-regression (MR-BRT) approach to adjust data from various methods, aligning them with those from gold standard methods (27). Data for dietary risk factors were obtained from a 24-h dietary recall survey, which reported food and nutrient consumption in grams per individual per day (14). The exposure estimates for each dietary risk, representing the average intake in grams per person per day for each nutrient, were modeled by age, sex, year, and location using a spatiotemporal Gaussian process regression (ST-GPR) frame work (28, 29). Detailed methodological specifications regarding MR-BRT and ST-GPR are provided in Supplementary material 1.

### 2.4 Socio-demographic index

SDI is regarded as a composite indicator of background social and economic conditions that influence health outcomes in each location, and a higher SDI indicates a better socioeconomic condition, which was calculated from the indices of total fertility rate for women under 25 years, average years of schooling for individuals aged 15 and older, and lag-distributed income per capita. The 204 countries and regions were categorized into five super-regions (high, high-middle, middle, low-middle, and low levels) (30, 31) based on the SDI. Furthermore, the world was further categorized into 21 geographic regions.

### 2.5 Statistical analyses

To evaluate the burden of T2D-related CKD associated with dietary risk factors, we utilized variables such as the number of deaths, DALYs, and their age-standardized rates (ASRs) to analyze among different groups by sex, age, year, location. Temporal changes in age-standardized mortality and DALY rates of T2D-related CKD attributable to dietary factors from 1990 to 2021 were analyzed using estimated annual percentage changes (EAPC). It was assumed that the natural logarithm of ASR follows a linear trend over time. Therefore, the EAPC was calculated using the formula: ASR = α + β × year + ε. The EAPC and its 95% confidence interval (CI) were derived from the formula: 100 × (exp(β) − 1). A linear regression analysis was conducted to explore the relationship between ASR, EAPC in ASR and SDI values, which were evaluated using Spearman’s rank correlation tests and visualized with Locally Weighted Scatterplot Smoothing (LOWESS) curves. All statistical analyses were performed using R software (version 4.0.3). A *p* value of less than 0.05 was regarded as statistically significant.

## 3 Results

### 3.1 Global trend in T2D-related CKD burden attributable to dietary risk factors

**Figure 1:**
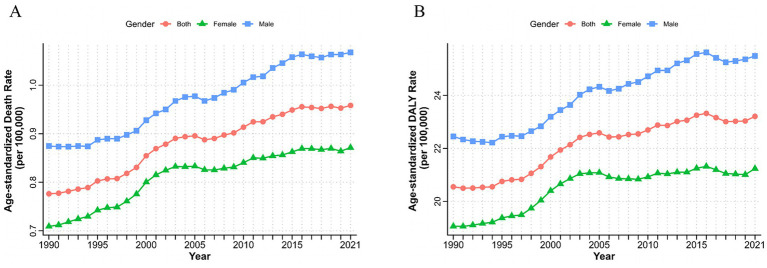
Burden of chronic kidney disease due to type 2 diabetes attributable to dietary risks by sex from 1990 to 2021. Age-standardized deaths **(A)** and DALYs **(B)** rate of chronic kidney disease due to type 2 diabetes attributable to dietary risks. DALYs, disability-adjusted life years.

From 1990 to 2021, ASMR and ASDR of T2D-related CKD attributable to dietary risk factors in global have increased by more than 2.5 times, exhibiting fluctuations but showing an overall upward trend (Figures 1A,B). In 2021, the global number of death and DALYs in T2D-related CKD attributable to dietary risks were estimated at 79,990 (95% *CI*: 32,730–128,880) and 1,999,210 (95% *CI*: 856,190–3,167,220) (Tables 1, 2). Global ASMR attributed to dietary risk factors in T2D-related CKD increased from 0.78 (95% *CI*: 0.31–1.23) per 100,000 persons in 1990 to 0.96 (95% *CI*: 0.40–1.54) per 100,000 persons in 2021 (Table 1), while the ASDR rose from 20.55 (95% *CI*: 8.42–32.26) per 100,000 persons in 1990 to 23.21 (95% *CI*: 9.95–36.61) per 100,000 persons in 2021 (Table 2). Furthermore, the overall burden of T2D-related CKD was on the rise. From 1990 to 2021, the EAPC of global ASMR and ASDR in T2D-related CKD attributed to dietary risk factors showed a gradual increase, with EAPCs of 0.76% (95% *CI*: 0.69–0.83%) and 0.47% (95% *CI*: 0.41–0.53%) for ASMR and ASDR, respectively (Tables 1, 2). Similar trends were observed across genders.

**Table 1:** Deaths and ASMR of T2D-related CKD burden attributable to dietary risk factors in 1990 and 2021 and the temporal trends from 1990 to 2021.

Characteristics	Death cases, No. ×10^3^ (95% *CI*)		ASMR per 100,000 people (95% *CI*)		EAPC of ASMR, % (95% *CI*)
	1990	2021	1990	2021	1990–2021
**Global**	27.23 (11.10–42.89)	79.99 (32.73–128.88)	0.78 (0.31–1.23)	0.96 (0.40–1.54)	0.76 (0.69–0.83)
**Sex**					
Male	13.11 (5.27–21.57)	39.19 (16.13–64.11)	0.87 (0.35–1.42)	1.07 (0.44–1.75)	0.77 (0.72–0.82)
Female	14.12 (5.83–22.25)	40.80 (16.96–65.21)	0.71 (0.29–1.14)	0.87 (0.36–1.39)	0.70 (0.60–0.79)
**SDI groups**					
High SDI	7.83 (3.58–11.80)	24.87 (10.72–38.33)	0.70 (0.32–1.07)	1.06 (0.45–1.61)	1.45 (1.36–1.54)
High-middle SDI	4.89 (1.86–8.06)	11.43 (4.58–18.95)	0.57 (0.22–0.93)	0.59 (0.24–0.97)	0.11 (0.04–0.18)
Middle SDI	7.92 (2.95–13.16)	25.47 (9.85–42.81)	0.94 (0.35–1.59)	1.03 (0.40–1.73)	0.42 (0.36–0.47)
Low-middle SDI	4.35 (1.72–7.19)	13.26 (5.43–22.59)	0.84 (0.32–1.40)	1.01 (0.41–1.72)	0.68 (0.65–0.72)
Low SDI	2.20 (0.89–3.66)	4.89 (1.97–8.49)	1.15 (0.46–1.92)	1.16 (0.44–2.02)	0.00 (−0.05 to 0.05)
**GBD region**					
East Asia	6.16 (1.84–10.78)	16.12 (5.18–28.75)	0.95 (0.30–1.67)	0.82 (0.26–1.43)	−0.57 (−0.70 to −0.44)
Southeast Asia	1.68 (0.52–3.13)	5.23 (1.55–10.12)	0.79 (0.25–1.48)	0.90 (0.27–1.74)	0.53 (0.48–0.58)
Oceania	0.03 (0.01–0.05)	0.08 (0.03–0.15)	1.13 (0.32–2.10)	1.38 (0.43–2.50)	0.56 (0.47–0.66)
Central Asia	0.10 (0.05–0.16)	0.31 (0.15–0.48)	0.22 (0.11–0.35)	0.41 (0.20–0.64)	1.34 (0.86–1.82)
Central Europe	0.49 (0.23–0.77)	0.68 (0.31–1.12)	0.35 (0.16–0.54)	0.29 (0.13–0.47)	−0.55 (−0.80 to −0.30)
Eastern Europe	0.48 (0.20–0.73)	0.90 (0.43–1.41)	0.17 (0.07–0.26)	0.25 (0.12–0.39)	0.81 (0.37–1.26)
High-income Asia Pacific	2.41 (1.22–3.57)	5.56 (2.41–8.76)	1.34 (0.68–1.99)	0.86 (0.37–1.31)	−1.54 (−1.65 to −1.43)
Australasia	0.04 (0.02–0.06)	0.14 (0.06–0.25)	0.18 (0.08–0.29)	0.24 (0.10–0.41)	1.68 (1.22–2.14)
Western Europe	2.79 (1.26–4.41)	5.75 (2.55–9.58)	0.46 (0.20–0.75)	0.46 (0.20–0.75)	0.46 (0.22–0.69)
Southern Latin America	0.63 (0.23–1.09)	1.00 (0.4–1.66)	1.44 (0.52–2.47)	1.11 (0.44–1.84)	−0.47 (−0.85 to −0.09)
High-income North America	2.67 (1.11–4.16)	12.93 (4.94–19.98)	0.74 (0.31–1.15)	1.90 (0.71–2.91)	3.18 (2.91–3.46)
Caribbean	0.32 (0.13–0.53)	0.92 (0.40–1.60)	1.32 (0.52–2.20)	1.70 (0.74–2.95)	1.37 (1.17–1.57)
Andean Latin America	0.29 (0.10–0.51)	1.15 (0.42–2.08)	1.55 (0.51–2.77)	2.01 (0.73–3.63)	0.95 (0.76–1.14)
Central Latin America	0.84 (0.36–1.41)	4.39 (1.89–7.26)	1.17 (0.49–1.99)	1.79 (0.76–2.97)	2.03 (1.51–2.55)
Tropical Latin America	1.09 (0.46–1.76)	3.94 (1.67–6.32)	1.38 (0.57–2.25)	1.57 (0.66–2.52)	0.40 (0.17–0.63)
North Africa and Middle East	1.23 (0.46–2.19)	3.01 (1.15–5.15)	0.86 (0.32–1.54)	0.73 (0.28–1.24)	−0.53 (−0.67 to −0.39)
South Asia	3.89 (1.62–6.62)	13.04 (5.23–23.15)	0.79 (0.32–1.34)	0.96 (0.37–1.68)	0.62 (0.53–0.71)
Central Sub-Saharan Africa	0.29 (0.10–0.50)	0.59 (0.21–1.07)	1.59 (0.55–2.76)	1.37 (0.45–2.54)	−0.76 (−0.87 to −0.66)
Eastern Sub-Saharan Africa	1.12 (0.45–1.96)	2.63 (1.11–4.47)	1.81 (0.70–3.15)	1.99 (0.81–3.37)	0.18 (0.13–0.23)
Southern Sub-Saharan Africa	0.12 (0.05–0.22)	0.34 (0.14–0.59)	0.52 (0.19–0.93)	0.67 (0.26–1.19)	1.27 (0.95–1.59)
Western Sub-Saharan Africa	0.58 (0.19–1.02)	1.24 (0.44–2.15)	0.80 (0.26–1.41)	0.77 (0.27–1.33)	−0.21 (−0.30 to −0.13)

ASMR, age-standardized mortality rate; T2D-related CKD, chronic kidney disease due to diabetes mellitus type 2; EAPC, estimated annual percentage change; CI, uncertainty interval; CI, confidence interval; SDI, socio-demographic index; GBD, Global Burden of Disease.

**Table 2:** DALYs and ASDR of T2D-related CKD burden attributable to dietary risk factors in 1990 and 2021 and the temporal trends from 1990 to 2021.

Characteristics	DALY cases, No. ×10^3^ (95% *CI*)		ASDR per 100,000 people (95% *CI*)		EAPC of ASDR, % (95% *CI*)
	1990	2021	1990	2021	1990–2021
**Global**	798.26 (322.75–1,247.38)	1,999.21 (856.19–3,167.22)	20.55 (8.42–32.26)	23.21 (9.95–36.61)	0.47 (0.41–0.53)
**Sex**					
Male	397.11 (162.06–642.07)	1,018.88 (430.05–1,628.74)	22.45 (9.26–36.27)	25.49 (10.72–40.90)	0.53 (0.48–0.58)
Female	401.15 (162.17–633.67)	980.33 (421.04–1,545.52)	19.06 (7.76–30.00)	21.24 (9.13–33.47)	0.37 (0.30–0.45)
**SDI groups**					
High SDI	219.66 (102.02–333.64)	541.20 (234.39–830.25)	19.92 (9.21–30.27)	26.48 (11.50–40.29)	1.05 (0.97–1.13)
High-middle SDI	155.75 (62.29–245.39)	289.16 (117.29–466.91)	16.11 (6.39–25.55)	14.70 (5.96–23.77)	−0.30 (−0.36 to −0.24)
Middle SDI	229.50 (84.92–377.62)	656.54 (265.08–1,054.87)	22.81 (8.40–37.40)	24.46 (9.87–39.17)	0.34 (0.27–0.40)
Low-middle SDI	129.91 (51.28–208.95)	374.36 (156.64–628.27)	21.66 (8.39–35.07)	25.88 (10.81–43.48)	0.66 (0.62–0.70)
Low SDI	62.65 (24.92–103.25)	136.37 (56.00–233.05)	28.19 (11.42–46.20)	27.41 (11.32–46.78)	−0.13 (−0.16 to −0.10)
**GBD region**					
East Asia	175.25 (50.36–311.38)	396.71 (123.41–701.90)	21.51 (6.33–37.83)	18.46 (5.75–32.68)	−0.45 (−0.59 to −0.31)
Southeast Asia	47.64 (14.38–84.81)	140.94 (44.68–259.24)	19.28 (6.13–34.36)	21.51 (6.85–39.70)	0.47 (0.41–0.52)
Oceania	0.86 (0.22–1.57)	2.40 (0.77–4.38)	28.15 (7.59–51.39)	31.79 (9.99–57.16)	0.33 (0.25–0.41)
Central Asia	7.36 (3.71–11.22)	14.22 (7.16–21.36)	15.57 (7.85–23.69)	16.80 (8.37–25.05)	−0.09 (−0.34 to 0.17)
Central Europe	17.85 (9.01–26.58)	21.05 (10.85–33.16)	11.99 (6.10–18.00)	9.62 (4.95–15.10)	−0.58 (−0.70 to −0.45)
Eastern Europe	30.71 (14.09–45.58)	31.97 (15.91–48.33)	10.99 (5.08–16.43)	9.00 (4.48–13.60)	−1.11 (−1.28 to −0.94)
High-income Asia Pacific	57.08 (29.92–84.08)	96.93 (43.80–145.72)	29.20 (15.28–43.02)	18.93 (8.98–28.32)	−1.37 (−1.51 to −1.23)
Australasia	1.73 (0.80–2.64)	4.36 (1.94–6.89)	7.47 (3.44–11.52)	8.37 (3.70–13.03)	0.60 (0.35–0.84)
Western Europe	81.56 (37.21–126.68)	117.70 (52.84–183.08)	14.04 (6.41–21.64)	11.89 (5.35–18.39)	−0.37 (−0.47 to −0.26)
Southern Latin America	14.94 (5.54–24.53)	21.15 (8.61–33.75)	32.57 (12.21–53.52)	24.26 (9.93–39.12)	−0.61 (−0.92 to −0.30)
High-income North America	79.22 (31.92–122.66)	298.15 (114.21–458.53)	23.00 (9.35–35.74)	47.82 (17.94–73.13)	2.53 (2.30–2.77)
Caribbean	8.11 (3.35–13.08)	21.57 (9.69–36.12)	31.48 (12.93–50.63)	39.96 (18.02–66.79)	1.28 (1.11–1.45)
Andean Latin America	6.71 (2.27–11.72)	25.76 (9.99–45.11)	33.58 (11.37–58.71)	43.69 (16.77–76.52)	0.95 (0.76–1.15)
Central Latin America	23.58 (10.20–37.51)	114.05 (51.69–183.13)	28.83 (12.59–46.01)	44.94 (20.14–71.41)	1.97 (1.48–2.45)
Tropical Latin America	31.21 (13.63–49.49)	95.99 (41.54–153.25)	34.39 (14.81–54.83)	37.19 (16.04–59.24)	0.15 (−0.09 to 0.38)
North Africa and Middle East	34.32 (13.05–59.04)	84.42 (31.87–141.58)	20.37 (7.78–35.35)	17.65 (6.78–29.90)	−0.48 (−0.55 to −0.41)
South Asia	121.19 (49.98–198.22)	377.97 (158.41–645.46)	21.25 (8.66–34.94)	25.33 (10.52–43.10)	0.61 (0.53–0.68)
Central Sub-Saharan Africa	8.48 (3.19–14.39)	17.81 (6.61–31.90)	38.50 (14.00–65.95)	32.62 (11.15–58.23)	−0.80 (−0.89 to −0.70)
Eastern Sub-Saharan Africa	28.03 (11.33–48.86)	63.95 (27.42–109.06)	39.33 (15.91–69.14)	40.98 (17.42–69.45)	0.00 (−0.05 to 0.05)
Southern Sub-Saharan Africa	4.62 (1.80–7.91)	11.66 (4.88–19.76)	16.67 (6.45–29.41)	19.57 (8.21–33.40)	0.78 (0.54–1.02)
Western Sub-Saharan Africa	17.81 (5.88–31.26)	40.44 (15.57–67.10)	20.69 (6.80–35.96)	20.18 (7.41–33.81)	−0.10 (−0.16 to −0.04)

T2D-related CKD, chronic kidney disease due to diabetes mellitus type 2; ASDR, Age-standardized DALY rate; DALY, disability-adjusted life year; CI, confidence interval; EAPC, estimated annual percentage change; SDI, socio-demographic index; GBD, Global Burden of Disease.

### 3.2 The burden of T2D-related CKD attributable to dietary risk factors by SDI levels

**Figure 2:**
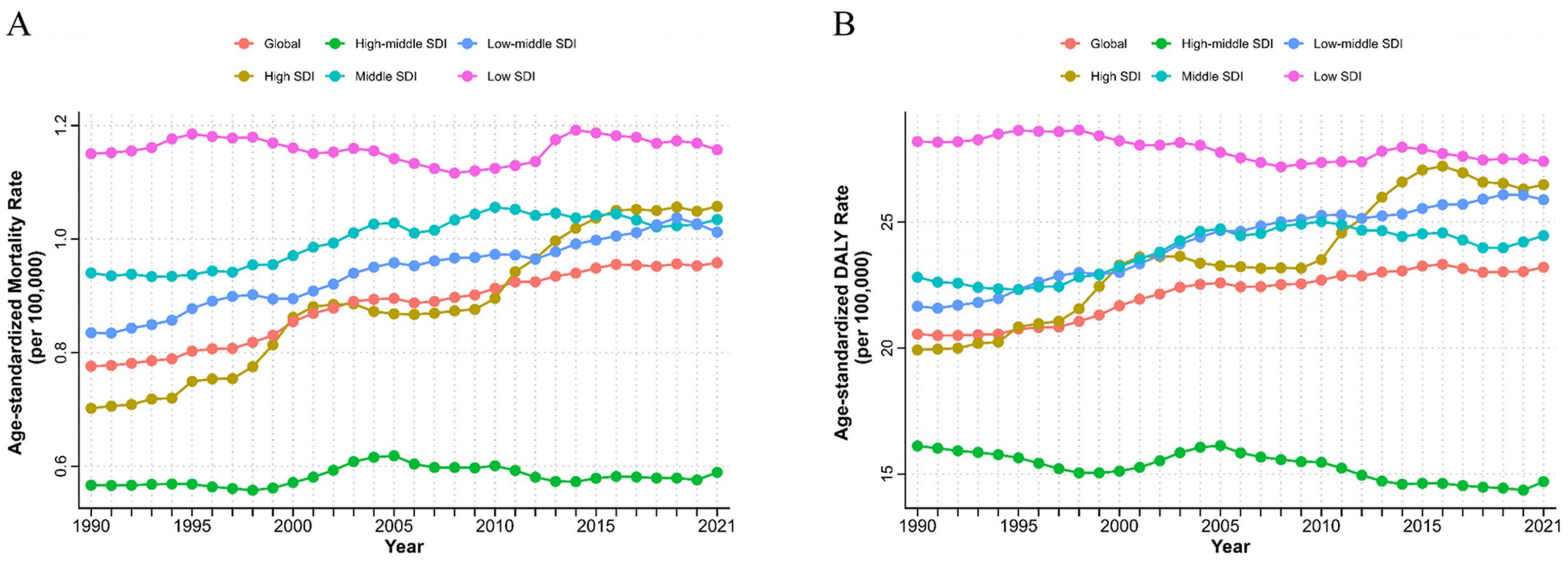
Burden of chronic kidney disease due to type 2 diabetes attributable to dietary risks with different SDI levels from 1990 to 2021. AMSR **(A)** and age-standardized DALYs rate **(B)** of T2D-related CKD attributable to dietary risks. SDI, Socio-demographic Index; ASMR, age-standardized mortality rate; DALYs, disability-adjusted life years; T2D-related CKD, chronic kidney disease due to type 2 diabetes.

At different SDI regional levels, high-middle SDI regions exhibited the lowest ASMR and ASDR for T2D-related CKD attributable to dietary risks, whereas low SDI regions had the highest ASMR and ASDR from 1990 to 2021. In 2021, the ASMR in high-middle SDI regions was 0.59 (95% *CI*: 0.24–0.97) per 100,000 persons, compared to 1.16 (95% *CI*: 0.44–2.02) per 100,000 persons in low SDI regions (Figure 2A; Table 1). The ASDR was 14.70 (95% *CI*: 5.96–23.77) per 100,000 persons in the high-middle SDI regions and 27.41 (95% *CI*: 11.32–46.78) per 100,000 persons in the low SDI regions (Figure 2B; Table 2). From 1990 to 2021, the ASMR of T2D-related CKD attributable to dietary risks generally showed an increasing trend across different SDI regions, with a highest EAPC in high SDI regions of 1.45% (95% *CI*: 1.36–1.54%) (Figure 2B; Table 1). Notably, the high SDI regions also experienced maximal increase in the ASDR, with an EAPC of 1.05% (95% *CI*: 0.97–1.13%), while the trend of high-middle and low SDI regions decreased (Figures 2A,B; Tables 1, 2). Detailed data on ASMR, ASDR and the EAPCs for T2D-related CKD attributable to dietary risks in various regions in 1990 and 2021, are presented in Figure 2 and Tables 1, 2.

### 3.3 Location burden in T2D-related CKD attributable to dietary risks

**Figure 3:**
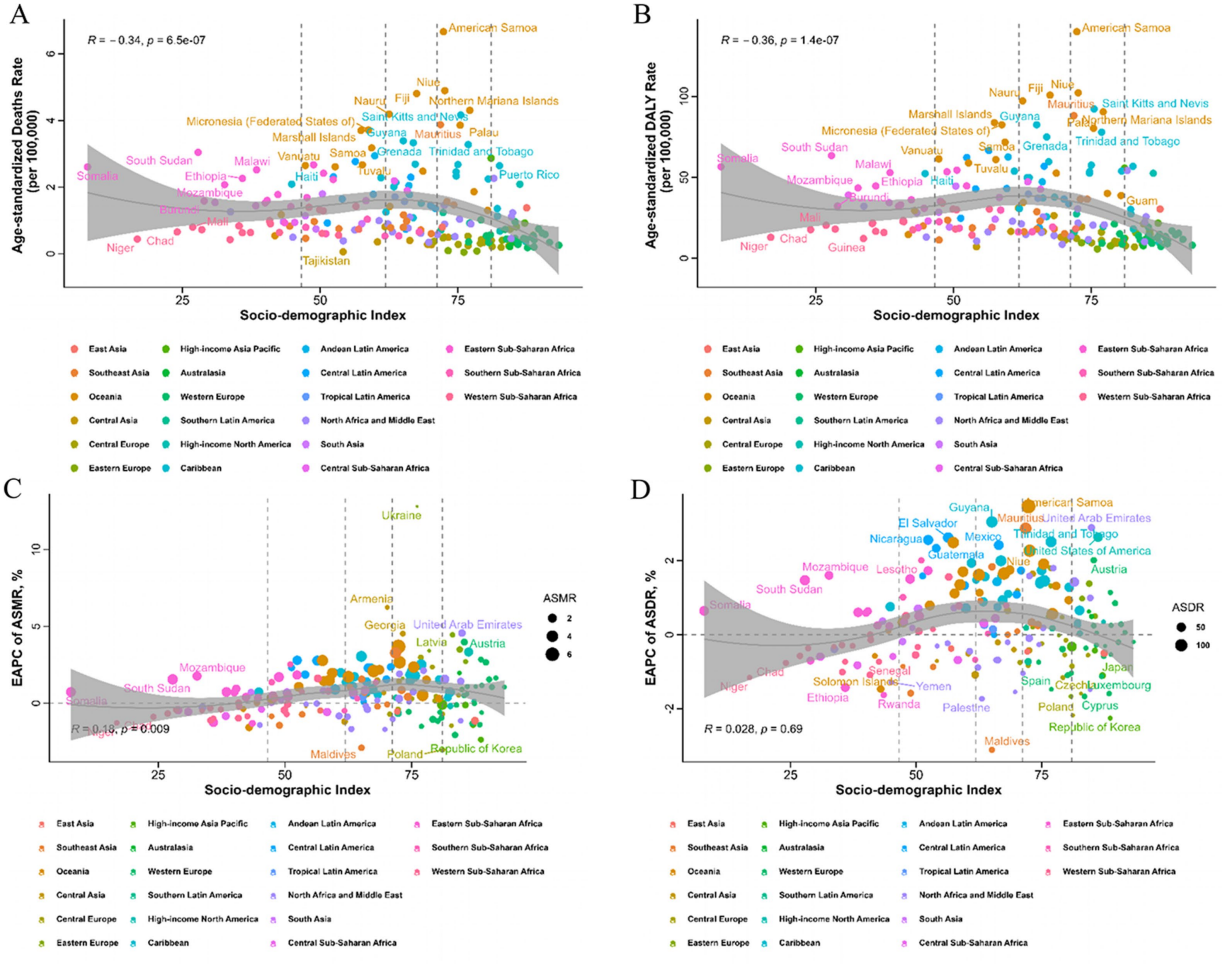
Location burden of chronic kidney disease due to type 2 diabetes attributable to dietary risks with different SDI levels. Correlation between ASMR **(A)** or ASDR **(B)** and countries of T2D-related CKD attributable to dietary risks in 2021. Correlation between EAPC of ASMR **(C)** or ASDR **(D)** and countries of T2D-related CKD attributable to dietary risks attributable to dietary risks from 1990 to 2021. Every dot represented a country or territory and its color implied the region that the country or territory located. SDI, Socio-demographic Index; ASMR, age-standardized mortality rate; ASDR, age-standardized DALYs rate; DALYs, disability-adjusted life years; T2D-related CKD, chronic kidney disease due to type 2 diabetes; EAPC, estimated annual percentage change; ASDR, age-standardized disability-adjusted life years rate.

At the regional and national levels, the burden of T2D-related CKD attributable to dietary factors exhibited substantial heterogeneity. In 2021, the East Asia region reported the highest burden of T2D-related CKD, with deaths of 16,120 (95% *CI*: 5,180–28,750) per 100,000 persons and DALYs of 396,710 (95%*CI*: 123,410–701,900) (Tables 1, 2). The top three regions with the highest ASMR in 2021 were Andean Latin America (2.01, 95%*CI*: 0.73–3.63 per 100,000 persons), Eastern Sub-Saharan Africa (1.99, 95%*CI*: 0.81–3.37 per 100,000 persons), and High-income North America (1.90, 95% *CI*: 0.71–2.91 per 100,000 persons) (Figure 3A; Table 1). And the regions with the highest ASDR in 2021 were High-income North America (47.82, 95% *CI*: 17.94–73.13 per 100,000 persons), Central Latin America (44.94, 95% *CI*: 20.14–71.41 per 100,000 persons), and Andean Latin America (43.69, 95% *CI*: 16.77–76.52 per 100,000 persons) (Figure 3B; Table 2). Among all countries, American Samoa bear the heaviest burden of T2D-related CKD attributable to dietary factors (Supplementary Figure 1). In 2021, the ASMR and ASDR were 6.66 (95% *CI*: 2.00–12.21) per 100,000 persons and 140.02 (95% *CI*: 43.91–257.50) per 100,000 persons, respectively (Supplementary Tables 1, 2).

From 1990 to 2021, the region with the most pronounced increase in the burden of T2D-related CKD attributable to dietary risks was High-income North America, with an EAPCs of 3.18% (95% *CI*: 2.91–3.46%) and 2.53% (95% *CI*: 2.30–2.77%) in ASMR and ASDR, respectively. In contrast, the region with the largest decrease was High-income Asia Pacific, with EAPCs of −1.54% (95% *CI*: −1.65% to −1.43%) and −1.37% (95% *CI*: −1.51% to −1.23%) in ASMR and ASDR, respectively (Figures 3A,B; Tables 1, 2). Among individual countries, Ukraine exhibited the highest increase in ASMR, with an EAPC of 12.82% (95% *CI*: 10.80–14.87%) (Figure 3C; Supplementary Table 1), while American Samoa had the highest growth trend in the ASDR, with an EAPC of 3.46% (95% *CI*: 3.20–3.73%) (Figure 3D; Supplementary Table 2). Detailed data on EAPCs for ASMR and ASDR are presented in Figure 3 and Tables 1, 2.

We analyzed the correlation between the burdens of T2D-related CKD attributable to dietary risks and SDI levels. In 2021, ASMR and ASDR were negatively correlated with SDI, with coefficients of *R* = −0.34 (*p* < 0.05) and *R* = −0.36 (*p* < 0.05), respectively (Figures 3A,B). From 1990 to 2021, the EAPC of ASMR across countries and regions was positively correlated with SDI, yielding a coefficient of 0.18 (*p* = 0.009). Whereas the EAPC of ASDR did not show significant correlation with SDI level (*R* = 0.028, *p* = 0.69) (Figure 3D).

### 3.4 Burden of T2D-related CKD attributable to dietary risk in different sexes and age groups

**Figure 4:**
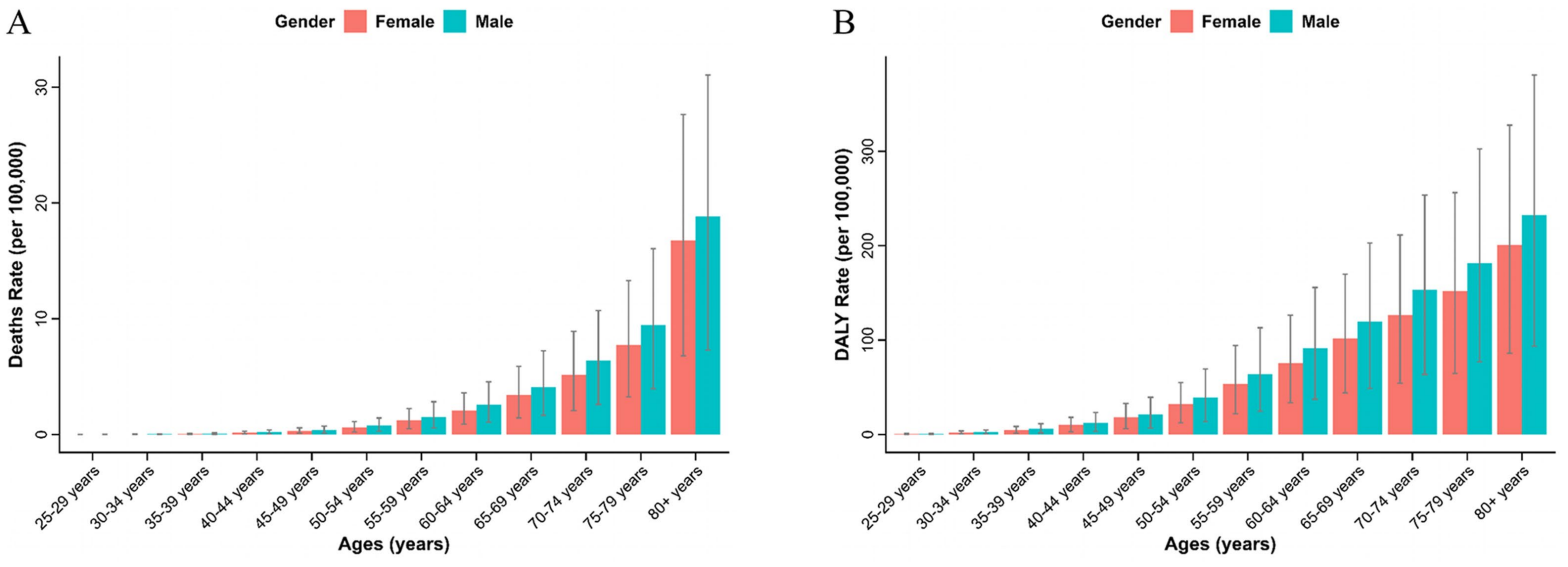
Chronic kidney disease due to type 2 diabetes attributable to dietary risk in different age groups in 2021. Mortality **(A)** and DALY **(B)** rates of chronic kidney disease due to diabetes mellitus. DALY, disability-adjusted life year.

From 1990 to 2021, both sexes exhibited upward trends consistent with the overall population. However, the burden of T2D-related CKD remained consistently higher in males compared to females. ASMR for males increased from 0.87 (95% *CI*: 0.35–1.42) per 100,000 persons in 1990 to 1.07 (95% *CI*: 0.44–1.75) per 100,000 persons in 2021, and for females, it rose from 0.71 (95% *CI*: 0.29–1.14) per 100,000 persons to 0.87 (95% *CI*: 0.36–1.39) per 100,000 persons over the same period (Table 1; Figure 1A). ASDR for males increased from 22.45 (95% *CI*: 9.26–36.27) to 25.49 (95% *CI*: 10.72–40.90) per 100,000 persons, and for females, from 19.06 (95% *CI*: 7.76–30.00) in 1990 to 21.24 (95% *CI*: 9.13–33.47) per 100,000 persons in 2021, respectively, resulting in a male-to-female ratio of approximately 1.2 (Figure 1B; Table 2). The EAPC for ASMR was 0.77% (95% *CI*: 0.72–0.82%) for males and 0.70% (95% *CI*: 0.60–0.79%) for females, and the EAPC for DALY was 0.53% (95% *CI*: 0.48–0.58%) and 0.37% (95% *CI*: 0.30–0.45%), respectively (Tables 1, 2). Male mortality and DALY rates in T2D-related CKD attributable to dietary risks were consistently higher across all age groups compared to females (Supplementary Figure 3). In 2021, the mortality and DALY rates in T2D-related CKD attributable to dietary risks peaked among males and females aged >80 years (Figures 4A,B), particularly concentrated in the age group >70 years, showing a sharp upward trend.

### 3.5 Detailed dietary risk factors contributed to the burden of T2D-related CKD

**Figure 5:**
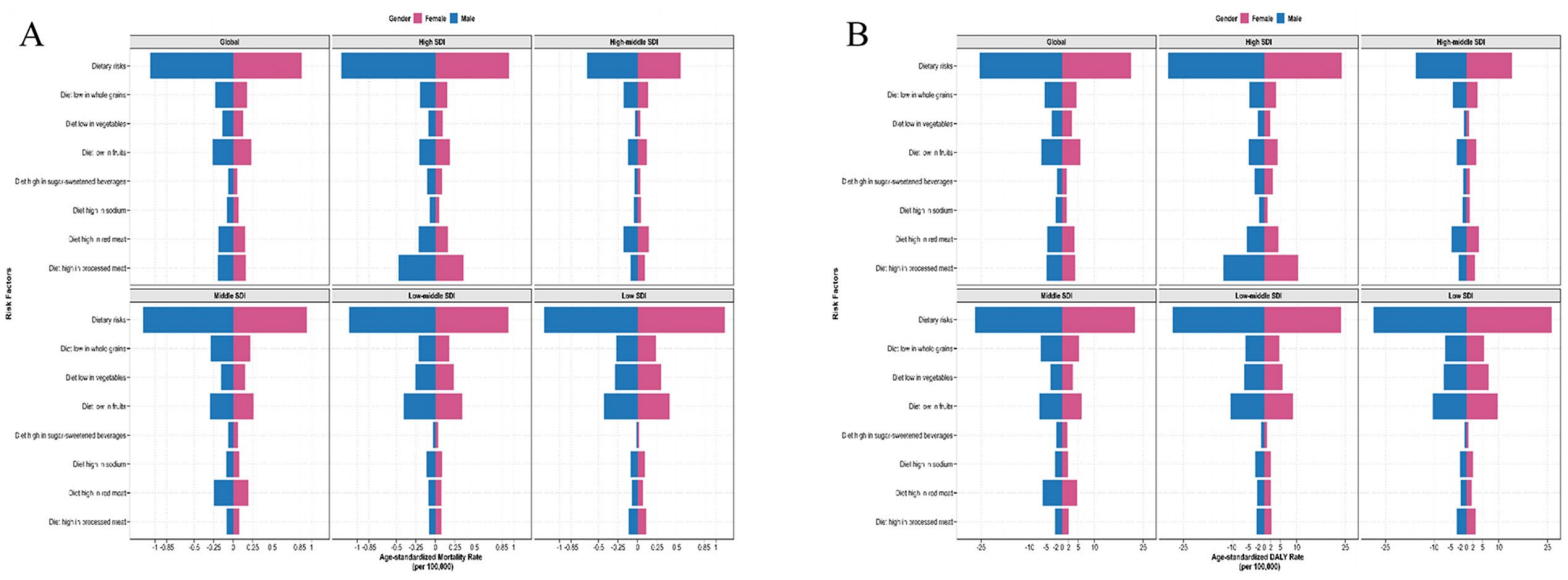
The detailed dietary risks burden for chronic kidney disease due to type 2 diabetes by sex with different SDI levels in 2021. ASMR **(A)** and ASDR **(B)** of detailed dietary risks factor of T2D-related CKD. SDI, Socio-demographic Index; ASMR, age-standardized mortality rate; DALY, disability-adjusted life years; T2D-related CKD, chronic kidney disease due to type 2 diabetes; ASDR, age-standardized disability-adjusted life years rate.

Globally, the top four dietary risk factors contributed to ASMR and ASDR related to T2D-related CKD were as follows: diet low in whole fruits, diet low in whole grains, diet high in processed meat, and diet high in red meat (Figures 4A,B; Table 3). Thereinto, diet low in fruits demonstrated the highest contribution, with an ASMR of 0.26 (95% *CI*: 0.09–0.48) per 100,000 persons and an ASDR of 6.54 (95% *CI*: 2.42–11.73) per 100,000 persons in 2021. From 1990 to 2021, the burden of all detailed dietary factors exhibited a significant upward trend and diet high in sugar-sweetened beverages had the highest growth rate, with an EAPC of 3.78% (95% *CI*: 3.66–3.89%) for ASMR and 3.25% (95% *CI*: 3.15–3.35%) for the ASDR (Table 3). The ASMR and ASDR of diet-induced T2D-related CKD attributable to these four dietary factors collectively exceed 80% (Supplementary Figure 2). Regardless of the specific detailed dietary factors, ASMR and ASDR were consistently higher in males than females (Figures 4A,B). At the SDI level, the attribution of ASMR and ASDR to detailed dietary factors showed a general similarity, yet high SDI regions exhibited a greater attribution to diet high in processed meat (Figure 5). Conversely, in low-middle and low SDI regions, the leading specific dietary risk factors that contributed to T2D-related CKD ranked as follows: diet low in whole fruits, diet low in vegetables, diet low in whole grains, and diet high in processed meat and red meat, emphasizing insufficient intake of healthy foods as a primary dietary risk (Figure 5). Globally, from 1990 to 2021, there was a consistent slow upward trend observed in both ASMR and ASDR. Diet low in whole fruits, diet low in whole grains, and diet high in processed meat and red meat consistently remained the predominant specific dietary risk factors contributing to T2D-related CKD (Supplementary Figure 2).

**Table 3:** Detailed dietary factors burden of T2D-related CKD attributable to dietary risks in 1990 and 2021, and the estimated annual percentage changes of death and DALY from 1990 to 2021.

Dietary risk	Age-standardized death and DALY rate per 100,000 people (95% *CI*)		EAPC of dietary risk, % (95% *CI*)
	1990	2021	1990–2021
Death			
Diet low in whole grains	0.12 (0.03–0.21)	0.21 (0.06–0.38)	1.88 (1.84–1.92)
Diet high in sodium	0.04 (0.00–0.14)	0.08 (0.00–0.32)	2.08 (1.98–2.18)
Diet low in fruits	0.15 (0.05–0.27)	0.26 (0.09–0.48)	1.74 (1.70–1.79)
Diet low in vegetables	0.08 (0.02–0.17)	0.14 (0.04–0.32)	2.07 (1.99–2.14)
Diet high in processed meat	0.08 (0.02–0.15)	0.18 (0.05–0.31)	2.75 (2.64–2.86)
Diet high in sugar-sweetened beverages	0.02 (0.01–0.03)	0.06 (0.03–0.09)	3.78 (3.66–3.89)
Diet high in red meat	0.08 (0.00–0.18)	0.18 (0.00–0.39)	2.83 (2.76–2.91)
DALY			
Diet low in whole grains	3.49 (0.92–6.29)	5.30 (1.37–9.59)	1.41 (1.37–1.46)
Diet high in sodium	1.07 (0.07–3.89)	1.87 (0.04–7.59)	1.71 (1.61–1.82)
Diet low in fruits	4.40 (1.64–7.74)	6.54 (2.42–11.73)	1.27 (1.23–1.31)
Diet low in vegetables	2.15 (0.53–4.41)	3.43 (0.91–7.51)	1.59 (1.50–1.69)
Diet high in processed meat	2.81 (0.72–5.00)	4.77 (1.21–8.29)	1.91 (1.81–2.01)
Diet high in sugar-sweetened beverages	0.67 (0.34–1.04)	1.69 (0.87–2.57)	3.25 (3.15–3.35)
Diet high in red meat	2.44 (0.00–5.16)	4.56 (0.00–9.80)	2.24 (2.16–2.32)

T2D-related CKD, chronic kidney disease due to diabetes mellitus type 2; DALY, disability-adjusted life year; CI, confidence interval; EAPC, estimated annual percentage change.

## 4 Discussion

Our analysis, for the first time, has provided a comprehensive overview of the global burden of T2D-related CKD attributable to detailed dietary risks in different sex, age and location groups from 1990 to 2021. Based on GBD 2021, this study revealed a consistent increase in the burden of T2D-related CKD over the past 30 years, with a notable contribution from dietary risk factors such as low fruit and whole grains intake, and high consumption of red and processed meats, particularly in males, the elderly, and the population in the low SDI regions. Our systematic analysis of T2D-related CKD attributable to dietary risks revealed a significant lack of awareness regarding the potential dangers of unhealthy dietary components in most countries. These findings provide valuable insights for the development of future food policies and programs that are flexible, integrated, and tailored to specific genders, age and geographic regions.

Over the past three decades, mortality and DALYs associated with T2D-related CKD attributable to dietary risks have increased significantly. Among these dietary risks, high sugar-sweetened beverage (SSBs) has the steepest increase in attributable burden globally from 1990 to 2021, which have been implicated in stimulating reward signals and contributing to ‘food addiction’, due to their rapid absorption and delivery of simple carbohydrates to the central nervous system (32). This rapid growth likely stems from widespread urbanization and economic development, which has increased the availability of SSBs, ultimately driving a global rise in sugar-sweetened beverage consumption (33). In addition, diet low in fruits, vegetables, and whole grains, alongside diet high in red and processed meats were also increased in attributable T2D-related CKD burden globally from 1990 to 2021. Despite the proposal of various dietary intervention measures, including mass media campaigns, food and menu labeling, food pricing strategies (subsidies and taxes), school procurement policies, and workplace wellness programs (34, 35), the efficacy of most of these interventions falls to achieve the levels required for optimal global diets (36–38). It is important that inexpensive, unhealthy ultra-processed foods face little taxation, while even when nutrition labels are understood, individuals have little incentive to opt for healthier alternatives (39, 40). Hence, our findings substantively align with the urgent imperatives outlined in Sustainable Development Goal 2 (41), emphasizing dietary risks are indeed crucial considerations in the management of future disease burden. Notably, American Samoa in Oceania shows the highest ASMR and age-standardized DALY rates attributable to dietary risks. This could be attributed to the higher prevalence of type 2 diabetes and kidney damage associated with filariasis in this region (42, 43). However, it does not directly indicate a correlation between diet and the high burden of T2D-related CKD in the region, and further research is needed to clarify this association in the aspect of cultural dietary norms, health access, or genetic susceptibility.

Different SDI regions exhibit divergent epidemiological patterns in dietary contributors to T2D-related CKD, with the most significantly increase observed in high SDI regions from 1990 to 2021. With sound medical systems, adequate medical resources and aging populations, T2D-related CKD patients are more likely to be reported in high SDI regions. Meanwhile, this can be explained by high excessive consumption of red and processed meat, leading to significant renal damage due to high concentrations of preservatives and other additives (44). Additionally, processed meat served as a primary source of dietary sodium (45), which has been proven to be significantly detrimental to T2D-related CKD, along with associations with the elevated blood pressure, proteinuria, obesity, insulin resistance, and metabolic syndrome (36). Our study has also revealed that low SDI regions consistently experience the highest disease burden due to dietary risks, as evidenced by the highest ASMR and ASDR from 1990 to 2021. In these regions, diets low in whole grains, vegetables, and fruits are identified as top three dietary risk factors, but high sugar-sweetened beverage has the lowest impact for T2D-related CKD. Other contributing factors included food security, unavailability of affordable whole grains, and insufficient public awareness on healthy dietary. The accessibility of cheap, ultra-processed foods, and unhealthy fats, coupled with the high cost of fresh fruits and vegetables, jointly contribute to the diet-related disease burden in low-SDI regions (46–49). Future intervention measures for reducing T2D-related CKD need to take into account the differences in dietary structure among regions.

Our study also found that from 1990 to 2021, the burden of T2D-related CKD attributable to dietary risks remained consistently higher in males compared to females across all age groups. Previous evidence indicated that hyperpalatable foods characterized by high salt content exhibited stronger addictive properties in male consumers, whereas females demonstrate greater dietary adherence to fruits and vegetables (50, 51). Our results also observed high greater red meat or sodium intake and lower fruits or grains among men globally. In addition, diagnostic criteria may also contribute to the imbalance of T2D-related CKD burden in between sexes. Clinical practice often employs a fixed body surface area (BSA) for GFR estimation, assuming equal kidney size between males and females (52). However, male kidneys tend to have larger volumes than female kidneys in reality (53). Even formulas like the CKD-EPI equation that incorporate gender as a variable tend to underestimated, GFR in males tends to be overestimated (54). This discrepancy suggests that the true burden of T2D-related CKD may be more severe in males compared to females.

From 1990 to 2021, the burden of T2D-related CKD was predominantly concentrated in individuals aged 70 and above. We observed an increasing trend in the burden of T2D-related CKD attributable to dietary risks with advancing age, especially in females. Given the longer life expectancy of females, consideration should be given to the natural decline in renal function among elderly women (55), which may have implications for their health outcomes. Moreover, it is believed that the protective effects of endogenous estrogen on renal function and structure contribute to the differences in T2D-related CKD prevalence between pre-menopausal and post-menopausal age groups (56). However, the average age of menopause is considered to be around 50 years (57), which does not align with the burden of T2D-related CKD being predominantly concentrated in individuals aged 70 and above in this study. This discrepancy may be explained by the slow progression of CKD (58).

This study still has certain limitations. The research data is derived from the GBD database, where the epidemiological evidence of causal relationships between dietary risks and disease endpoints largely comes from observational studies, which typically have weaker evidence strength compared to randomized controlled trials. Furthermore, data on various dietary risk factors originate from different sources and may not necessarily adhere to uniform assessment standards, which contribute to statistical uncertainties in our estimates of dietary risk exposure. Additionally, when estimating the dietary burden of T2D-related CKD, dietary factors are assumed to be independently distributed across each analytic unit. However, processed meat consumption often correlates with low fruit intake, which may oversimplify real-world dietary patterns. Finally, CKD staging significantly impacts the analysis of T2D-related CKD burden attributable to dietary risk factors, but there is a paucity of such data in the GBD database. These will require us to conduct newer, region-specific cohort studies or dietary assessments with sensitivity analysis or co-linearity in the future to verify current findings.

In conclusion, our study found an increasing global burden of T2D-related CKD attributable to dietary risks underscores the urgent need for targeted public health interventions focused on enhancing dietary quality. Our analysis offers a comprehensive, multidimensional assessment of the impact of dietary risks on T2D-related CKD. Researches and policy initiatives addressing specific dietary components, as well as focusing on men, the elderly, and low-SDI regions, could be highly effective to reduce the burden. For the aim of SDGs, collaborative efforts across sectors and stakeholders, including ministries of heath, agriculture, education and commerce will be essential to addressing the complex challenges posed by T2D-related CKD.

## Data Availability

Publicly available datasets were analyzed in this study. This data can be found at: https://vizhub.healthdata.org/gbd-results/.
