# Supplementary material for: Systematic analysis of the burden of chronic kidney disease due to type 2 diabetes attributable to dietary risks based on the global burden of disease study 2021
Source: Front Nutr. 2025 May 21;12:1572610. doi: 10.3389/fnut.2025.1572610 (PMC12133548; doi:10.3389/fnut.2025.1572610)
Supplement: Supplementary file 1 [file Table_1.docx]

**Supplementary Material 1 MR-BRT and ST-GPR**

MR-BRT (Meta-Regression—Bayesian, Regularized, Trimmed) is an advanced meta-analytic tool that harmonizes heterogeneous epidemiological data by combining Bayesian priors with regularization techniques to adjust for study-specific biases (e.g., measurement methods), automatically trimming outliers through iterative algorithms to produce robust exposure-response relationships.

ST-GPR (Spatio-Temporal Gaussian Process Regression) is a Bayesian modeling framework that generates continuous spatiotemporal estimates by integrating Gaussian process smoothing with covariate effects, enabling prediction of missing values while quantifying uncertainty through 95% UIs.

**Table S1 ASMR of T2D-related CKD attributable to dietary risks in 204 countries and territories, in 1990 and 2021, and the estimated annual percentage changes of DALY from 1990 to 2021**.

| **location_name** | **ASMR per 100,000 people**  **(95% *CI*)** | | **EAPC of DALY, % (95% *CI*)** |
| --- | --- | --- | --- |
|  | **1990** | **2021** | **1990-2021** |
| East Asia | 0.95 (0.30 to 1.67) | 0.82 (0.26 to 1.43) | -0.57 (-0.70 to -0.44) |
| Southeast Asia | 0.79 (0.25 to 1.48) | 0.90 (0.27 to 1.74) | 0.53 (0.48 to 0.58) |
| Oceania | 1.13 (0.32 to 2.10) | 1.38 (0.43 to 2.50) | 0.56 (0.47 to 0.66) |
| Central Asia | 0.22 (0.11 to 0.35) | 0.41 (0.20 to 0.64) | 1.34 (0.86 to 1.82) |
| Central Europe | 0.35 (0.16 to 0.54) | 0.29 (0.13 to 0.47) | -0.55 (-0.80 to -0.30) |
| Eastern Europe | 0.17 (0.07 to 0.26) | 0.25 (0.12 to 0.39) | 0.81 (0.37 to 1.26) |
| High-income Asia Pacific | 1.34 (0.68 to 1.99) | 0.86 (0.37 to 1.31) | -1.54 (-1.65 to -1.43) |
| Australasia | 0.18 (0.08 to 0.29) | 0.24 (0.10 to 0.41) | 1.68 (1.22 to 2.14) |
| Western Europe | 0.46 (0.20 to 0.75) | 0.46 (0.20 to 0.75) | 0.46 (0.22 to 0.69) |
| Southern Latin America | 1.44 (0.52 to 2.47) | 1.11 (0.44 to 1.84) | -0.47 (-0.85 to -0.09) |
| High-income North America | 0.74 (0.31 to 1.15) | 1.90 (0.71 to 2.91) | 3.18 (2.91 to 3.46) |
| Caribbean | 1.32 (0.52 to 2.20) | 1.70 (0.74 to 2.95) | 1.37 (1.17 to 1.57) |
| Andean Latin America | 1.55 (0.51 to 2.77) | 2.01 (0.73 to 3.63) | 0.95 (0.76 to 1.14) |
| Central Latin America | 1.17 (0.49 to 1.99) | 1.79 (0.76 to 2.97) | 2.03 (1.51 to 2.55) |
| Tropical Latin America | 1.38 (0.57 to 2.25) | 1.57 (0.66 to 2.52) | 0.40 (0.17 to 0.63) |
| North Africa and Middle East | 0.86 (0.32 to 1.54) | 0.73 (0.28 to 1.24) | -0.53 (-0.67 to -0.39) |
| South Asia | 0.79 (0.32 to 1.34) | 0.96 (0.37 to 1.68) | 0.62 (0.53 to 0.71) |
| Central Sub-Saharan Africa | 1.59 (0.55 to 2.76) | 1.37 (0.45 to 2.54) | -0.76 (-0.87 to -0.66) |
| Eastern Sub-Saharan Africa | 1.81 (0.70 to 3.15) | 1.99 (0.81 to 3.37) | 0.18 (0.13 to 0.23) |
| Southern Sub-Saharan Africa | 0.52 (0.19 to 0.93) | 0.67 (0.26 to 1.19) | 1.27 (0.95 to 1.59) |
| Western Sub-Saharan Africa | 0.80 (0.26 to 1.41) | 0.77 (0.27 to 1.33) | -0.21 (-0.30 to -0.13) |
| Afghanistan | 1.39 (0.44 to 2.82) | 1.26 (0.41 to 2.98) | -0.27 (-0.43 to -0.11) |
| Albania | 0.55 (0.27 to 0.86) | 0.37 (0.17 to 0.63) | -0.71 (-0.95 to -0.48) |
| Algeria | 0.82 (0.28 to 1.64) | 0.77 (0.26 to 1.50) | -0.19 (-0.48 to 0.10) |
| American Samoa | 2.30 (0.72 to 4.18) | 6.66 (2.00 to 12.21) | 3.68 (3.39 to 3.97) |
| Andorra | 0.80 (0.33 to 1.40) | 0.46 (0.20 to 0.81) | -1.32 (-1.55 to -1.09) |
| Angola | 0.98 (0.32 to 1.86) | 0.94 (0.29 to 1.87) | -0.40 (-0.54 to -0.27) |
| Antigua and Barbuda | 1.73 (0.65 to 2.95) | 2.68 (1.05 to 4.92) | 2.05 (1.76 to 2.35) |
| Argentina | 1.71 (0.60 to 3.03) | 1.24 (0.47 to 2.08) | -0.65 (-1.03 to -0.27) |
| Armenia | 0.03 (0.01 to 0.05) | 0.27 (0.12 to 0.47) | 6.24 (5.01 to 7.48) |
| Australia | 0.16 (0.07 to 0.26) | 0.22 (0.09 to 0.38) | 1.96 (1.53 to 2.40) |
| Austria | 0.42 (0.18 to 0.69) | 0.96 (0.43 to 1.45) | 3.98 (3.24 to 4.72) |
| Azerbaijan | 0.23 (0.11 to 0.37) | 0.28 (0.13 to 0.48) | 0.68 (0.36 to 1.01) |
| Bahamas | 1.69 (0.70 to 2.78) | 2.24 (0.91 to 3.88) | 1.52 (1.27 to 1.78) |
| Bahrain | 0.75 (0.29 to 1.35) | 1.04 (0.39 to 1.92) | 0.73 (0.44 to 1.02) |
| Bangladesh | 0.74 (0.27 to 1.34) | 0.74 (0.27 to 1.34) | 0.21 (0.12 to 0.29) |
| Barbados | 1.74 (0.70 to 2.87) | 2.46 (0.94 to 4.32) | 1.84 (1.47 to 2.21) |
| Belarus | 0.03 (0.01 to 0.05) | 0.08 (0.04 to 0.13) | 3.42 (2.61 to 4.23) |
| Belgium | 0.52 (0.22 to 0.85) | 0.46 (0.19 to 0.77) | 0.50 (0.02 to 0.99) |
| Belize | 1.40 (0.54 to 2.42) | 2.28 (0.91 to 4.03) | 1.98 (1.51 to 2.46) |
| Benin | 0.71 (0.21 to 1.30) | 0.64 (0.20 to 1.21) | -0.41 (-0.50 to -0.32) |
| Bermuda | 1.45 (0.56 to 2.38) | 1.36 (0.51 to 2.34) | 0.37 (0.06 to 0.67) |
| Bhutan | 1.16 (0.42 to 2.07) | 1.14 (0.42 to 2.18) | 0.06 (-0.03 to 0.15) |
| Bolivia (Plurinational State of) | 2.44 (0.82 to 4.32) | 2.94 (0.98 to 5.59) | 0.90 (0.79 to 1.00) |
| Bosnia and Herzegovina | 0.53 (0.26 to 0.85) | 0.48 (0.21 to 0.84) | -0.41 (-0.74 to -0.09) |
| Botswana | 0.50 (0.17 to 0.96) | 0.54 (0.17 to 1.04) | 0.30 (0.08 to 0.51) |
| Brazil | 1.38 (0.57 to 2.25) | 1.57 (0.66 to 2.52) | 0.40 (0.17 to 0.63) |
| Brunei Darussalam | 3.46 (1.30 to 6.35) | 2.87 (1.02 to 5.39) | -0.10 (-0.33 to 0.13) |
| Bulgaria | 0.27 (0.13 to 0.42) | 0.53 (0.24 to 0.87) | 2.98 (2.53 to 3.43) |
| Burkina Faso | 0.82 (0.24 to 1.52) | 0.72 (0.22 to 1.30) | -0.42 (-0.53 to -0.31) |
| Burundi | 1.60 (0.58 to 3.04) | 1.58 (0.54 to 3.17) | -0.46 (-0.61 to -0.30) |
| Cabo Verde | 0.39 (0.14 to 0.69) | 0.56 (0.16 to 1.12) | 0.73 (0.40 to 1.07) |
| Cambodia | 0.56 (0.14 to 1.09) | 0.46 (0.12 to 0.94) | -0.86 (-1.17 to -0.54) |
| Cameroon | 1.32 (0.37 to 2.50) | 1.39 (0.50 to 2.64) | 0.32 (0.03 to 0.61) |
| Canada | 0.39 (0.13 to 0.67) | 0.36 (0.13 to 0.62) | 0.12 (-0.19 to 0.43) |
| Central African Republic | 1.82 (0.64 to 3.33) | 1.54 (0.53 to 2.88) | -0.56 (-0.64 to -0.48) |
| Chad | 0.81 (0.24 to 1.55) | 0.65 (0.17 to 1.26) | -0.89 (-1.05 to -0.72) |
| Chile | 0.85 (0.32 to 1.38) | 0.92 (0.40 to 1.53) | 0.66 (0.17 to 1.16) |
| China | 0.93 (0.29 to 1.62) | 0.80 (0.26 to 1.41) | -0.56 (-0.69 to -0.42) |
| Colombia | 0.83 (0.33 to 1.42) | 0.74 (0.27 to 1.30) | 0.08 (-0.14 to 0.30) |
| Comoros | 1.39 (0.49 to 2.69) | 1.69 (0.57 to 3.30) | 0.51 (0.44 to 0.57) |
| Congo | 1.81 (0.60 to 3.29) | 1.59 (0.51 to 3.13) | -0.57 (-0.69 to -0.46) |
| Cook Islands | 1.18 (0.33 to 2.03) | 1.86 (0.57 to 3.48) | 1.52 (1.41 to 1.63) |
| Costa Rica | 0.94 (0.41 to 1.53) | 1.45 (0.56 to 2.49) | 1.55 (1.15 to 1.95) |
| Croatia | 0.32 (0.13 to 0.51) | 0.38 (0.15 to 0.70) | 0.44 (0.10 to 0.78) |
| Cuba | 0.69 (0.29 to 1.13) | 1.08 (0.48 to 1.78) | 2.13 (1.81 to 2.46) |
| Cyprus | 1.35 (0.55 to 2.41) | 0.73 (0.30 to 1.27) | -2.07 (-2.38 to -1.76) |
| Czechia | 0.30 (0.14 to 0.49) | 0.19 (0.07 to 0.34) | -1.46 (-1.68 to -1.24) |
| Côte d'Ivoire | 0.80 (0.25 to 1.41) | 0.64 (0.23 to 1.15) | -0.85 (-0.94 to -0.76) |
| Democratic People's Republic of Korea | 0.89 (0.23 to 1.66) | 0.74 (0.18 to 1.41) | -0.48 (-0.63 to -0.32) |
| Democratic Republic of the Congo | 1.71 (0.59 to 3.04) | 1.44 (0.45 to 2.67) | -0.84 (-0.95 to -0.73) |
| Denmark | 0.34 (0.16 to 0.53) | 0.75 (0.37 to 1.17) | 2.66 (2.34 to 2.99) |
| Djibouti | 1.58 (0.58 to 2.98) | 2.67 (0.99 to 4.80) | 1.70 (1.53 to 1.87) |
| Dominica | 1.60 (0.63 to 2.73) | 2.33 (0.93 to 4.20) | 1.46 (1.38 to 1.54) |
| Dominican Republic | 1.06 (0.38 to 1.90) | 1.25 (0.42 to 2.36) | 1.37 (1.06 to 1.69) |
| Ecuador | 1.37 (0.47 to 2.38) | 2.05 (0.75 to 3.80) | 1.40 (0.51 to 2.30) |
| Egypt | 1.28 (0.39 to 2.37) | 0.99 (0.38 to 1.81) | -0.83 (-0.91 to -0.75) |
| El Salvador | 1.18 (0.44 to 2.09) | 2.77 (1.08 to 5.18) | 2.77 (2.22 to 3.32) |
| Equatorial Guinea | 1.60 (0.53 to 2.72) | 1.89 (0.64 to 3.69) | 0.85 (0.43 to 1.28) |
| Eritrea | 1.21 (0.41 to 2.60) | 1.60 (0.52 to 3.43) | 0.94 (0.86 to 1.02) |
| Estonia | 0.22 (0.09 to 0.35) | 0.74 (0.33 to 1.18) | 3.50 (3.03 to 3.96) |
| Eswatini | 0.87 (0.26 to 1.53) | 1.03 (0.37 to 2.05) | 0.80 (0.23 to 1.36) |
| Ethiopia | 2.98 (0.77 to 5.61) | 2.26 (0.75 to 4.11) | -1.25 (-1.38 to -1.13) |
| Fiji | 2.18 (0.64 to 4.08) | 4.81 (1.61 to 8.95) | 2.09 (1.67 to 2.52) |
| Finland | 0.30 (0.16 to 0.45) | 0.35 (0.18 to 0.52) | 1.08 (0.87 to 1.30) |
| France | 0.53 (0.22 to 0.87) | 0.44 (0.19 to 0.69) | -0.11 (-0.48 to 0.25) |
| Gabon | 1.70 (0.56 to 3.24) | 2.19 (0.67 to 4.10) | 0.71 (0.51 to 0.91) |
| Gambia | 0.91 (0.27 to 1.64) | 0.90 (0.26 to 1.79) | -0.21 (-0.35 to -0.08) |
| Georgia | 0.13 (0.06 to 0.22) | 0.49 (0.26 to 0.79) | 4.53 (3.74 to 5.32) |
| Germany | 0.50 (0.24 to 0.77) | 0.62 (0.28 to 1.05) | 1.55 (1.03 to 2.06) |
| Ghana | 0.97 (0.31 to 1.75) | 1.42 (0.51 to 2.48) | 1.61 (1.33 to 1.89) |
| Greece | 1.15 (0.47 to 1.97) | 1.02 (0.45 to 1.63) | -1.05 (-2.00 to -0.09) |
| Greenland | 0.84 (0.29 to 1.46) | 0.68 (0.25 to 1.19) | -0.19 (-0.36 to -0.02) |
| Grenada | 1.94 (0.74 to 3.56) | 3.33 (1.21 to 6.15) | 2.24 (2.00 to 2.49) |
| Guam | 1.62 (0.52 to 2.82) | 1.30 (0.42 to 2.34) | 0.30 (-0.12 to 0.72) |
| Guatemala | 1.27 (0.44 to 2.21) | 1.65 (0.64 to 3.18) | 1.86 (1.31 to 2.42) |
| Guinea | 0.52 (0.14 to 1.00) | 0.42 (0.12 to 0.82) | -0.48 (-0.59 to -0.37) |
| Guinea-Bissau | 1.15 (0.36 to 2.13) | 0.83 (0.25 to 1.54) | -1.12 (-1.17 to -1.06) |
| Guyana | 1.87 (0.68 to 3.43) | 3.39 (1.18 to 6.51) | 3.05 (2.65 to 3.45) |
| Haiti | 1.82 (0.66 to 3.80) | 2.09 (0.66 to 4.86) | 0.73 (0.63 to 0.83) |
| Honduras | 0.49 (0.19 to 0.89) | 0.92 (0.33 to 1.79) | 2.31 (2.04 to 2.58) |
| Hungary | 0.22 (0.11 to 0.33) | 0.31 (0.13 to 0.51) | 2.35 (1.84 to 2.87) |
| Iceland | 0.16 (0.06 to 0.26) | 0.18 (0.07 to 0.30) | 1.08 (0.81 to 1.35) |
| India | 0.71 (0.29 to 1.22) | 0.90 (0.35 to 1.63) | 0.82 (0.71 to 0.93) |
| Indonesia | 0.79 (0.22 to 1.62) | 0.98 (0.29 to 2.17) | 0.71 (0.62 to 0.79) |
| Iran (Islamic Republic of) | 0.55 (0.18 to 1.03) | 0.35 (0.12 to 0.61) | -1.70 (-1.91 to -1.49) |
| Iraq | 1.15 (0.44 to 2.05) | 1.10 (0.39 to 2.20) | -0.41 (-0.65 to -0.16) |
| Ireland | 0.54 (0.26 to 0.86) | 0.44 (0.20 to 0.74) | -0.01 (-0.22 to 0.19) |
| Israel | 0.95 (0.40 to 1.57) | 0.57 (0.23 to 0.97) | -0.95 (-1.51 to -0.39) |
| Italy | 0.41 (0.18 to 0.68) | 0.34 (0.14 to 0.61) | -0.59 (-0.72 to -0.46) |
| Jamaica | 1.11 (0.38 to 1.97) | 1.51 (0.60 to 2.91) | 0.55 (-0.10 to 1.20) |
| Japan | 1.38 (0.70 to 2.04) | 0.92 (0.42 to 1.41) | -1.38 (-1.51 to -1.26) |
| Jordan | 1.28 (0.46 to 2.30) | 1.45 (0.49 to 2.47) | 0.30 (-0.09 to 0.69) |
| Kazakhstan | 0.27 (0.14 to 0.41) | 0.50 (0.24 to 0.77) | 1.43 (1.05 to 1.81) |
| Kenya | 1.40 (0.56 to 2.57) | 2.23 (0.89 to 3.90) | 1.83 (1.72 to 1.95) |
| Kiribati | 1.87 (0.45 to 3.26) | 2.61 (0.55 to 5.19) | 1.07 (0.89 to 1.25) |
| Kuwait | 0.71 (0.24 to 1.28) | 0.46 (0.16 to 0.85) | -1.14 (-1.40 to -0.88) |
| Kyrgyzstan | 0.16 (0.07 to 0.26) | 0.36 (0.15 to 0.59) | 0.94 (0.02 to 1.86) |
| Lao People's Democratic Republic | 1.43 (0.39 to 2.78) | 0.97 (0.26 to 1.99) | -1.44 (-1.57 to -1.31) |
| Latvia | 0.12 (0.05 to 0.19) | 0.41 (0.20 to 0.66) | 4.45 (4.00 to 4.90) |
| Lebanon | 0.53 (0.18 to 0.97) | 0.52 (0.18 to 0.94) | 0.10 (-0.15 to 0.36) |
| Lesotho | 0.35 (0.10 to 0.68) | 0.60 (0.20 to 1.09) | 2.53 (2.09 to 2.96) |
| Liberia | 1.00 (0.30 to 1.78) | 0.83 (0.25 to 1.64) | -0.33 (-0.65 to 0.00) |
| Libya | 0.61 (0.22 to 1.09) | 0.89 (0.33 to 1.72) | 1.91 (1.71 to 2.12) |
| Lithuania | 0.09 (0.04 to 0.14) | 0.31 (0.14 to 0.48) | 2.71 (2.11 to 3.31) |
| Luxembourg | 0.79 (0.34 to 1.25) | 0.49 (0.22 to 0.80) | -1.40 (-1.78 to -1.02) |
| Madagascar | 1.26 (0.43 to 2.29) | 1.53 (0.52 to 2.83) | 0.71 (0.64 to 0.78) |
| Malawi | 1.91 (0.69 to 3.48) | 2.52 (0.98 to 4.61) | 0.73 (0.50 to 0.97) |
| Malaysia | 1.32 (0.41 to 2.46) | 1.47 (0.42 to 2.78) | 0.42 (0.15 to 0.69) |
| Maldives | 1.73 (0.54 to 3.36) | 0.79 (0.21 to 1.63) | -2.90 (-3.13 to -2.67) |
| Mali | 1.03 (0.31 to 1.88) | 0.80 (0.24 to 1.50) | -0.67 (-0.78 to -0.57) |
| Malta | 0.80 (0.35 to 1.29) | 0.54 (0.23 to 0.90) | -1.16 (-1.59 to -0.72) |
| Marshall Islands | 1.68 (0.33 to 3.68) | 3.70 (0.46 to 11.12) | 2.78 (2.63 to 2.94) |
| Mauritania | 1.39 (0.42 to 2.60) | 1.32 (0.46 to 2.41) | -0.45 (-0.60 to -0.31) |
| Mauritius | 1.61 (0.44 to 2.94) | 3.87 (1.28 to 6.70) | 3.28 (2.81 to 3.75) |
| Mexico | 1.48 (0.64 to 2.52) | 2.30 (1.02 to 3.71) | 2.30 (1.60 to 3.00) |
| Micronesia (Federated States of) | 2.51 (0.56 to 4.95) | 3.71 (0.81 to 7.31) | 1.49 (1.28 to 1.70) |
| Monaco | 0.45 (0.19 to 0.74) | 0.58 (0.24 to 0.97) | 1.05 (0.74 to 1.36) |
| Mongolia | 1.01 (0.49 to 1.61) | 0.81 (0.41 to 1.28) | -1.20 (-1.37 to -1.02) |
| Montenegro | 0.34 (0.13 to 0.59) | 0.34 (0.12 to 0.64) | 0.16 (-0.08 to 0.39) |
| Morocco | 0.74 (0.29 to 1.50) | 0.71 (0.30 to 1.21) | -0.02 (-0.34 to 0.30) |
| Mozambique | 1.36 (0.50 to 2.72) | 2.07 (0.69 to 3.79) | 1.77 (1.59 to 1.94) |
| Myanmar | 0.90 (0.22 to 1.92) | 0.75 (0.21 to 1.55) | -0.73 (-0.79 to -0.66) |
| Namibia | 0.57 (0.19 to 1.04) | 0.53 (0.17 to 0.97) | -0.49 (-0.82 to -0.16) |
| Nauru | 2.36 (0.66 to 4.94) | 4.19 (1.18 to 8.77) | 1.72 (1.60 to 1.84) |
| Nepal | 0.84 (0.32 to 1.49) | 0.96 (0.33 to 1.72) | 0.71 (0.53 to 0.89) |
| Netherlands | 0.28 (0.12 to 0.47) | 0.42 (0.19 to 0.66) | 1.16 (0.60 to 1.71) |
| New Zealand | 0.29 (0.13 to 0.46) | 0.36 (0.15 to 0.57) | 0.89 (0.20 to 1.59) |
| Nicaragua | 1.19 (0.45 to 2.25) | 2.31 (0.81 to 4.30) | 2.79 (2.32 to 3.26) |
| Niger | 0.65 (0.21 to 1.23) | 0.44 (0.15 to 0.89) | -1.28 (-1.35 to -1.22) |
| Nigeria | 0.68 (0.22 to 1.19) | 0.61 (0.21 to 1.09) | -0.71 (-0.98 to -0.44) |
| Niue | 2.19 (0.70 to 4.22) | 4.89 (1.31 to 10.74) | 2.66 (2.55 to 2.78) |
| North Macedonia | 0.26 (0.12 to 0.43) | 0.28 (0.11 to 0.53) | 0.29 (-0.23 to 0.82) |
| Northern Mariana Islands | 3.76 (1.19 to 6.43) | 4.30 (1.38 to 7.69) | 0.52 (0.26 to 0.78) |
| Norway | 0.21 (0.09 to 0.33) | 0.34 (0.14 to 0.56) | 1.64 (1.14 to 2.15) |
| Oman | 0.61 (0.17 to 1.19) | 0.89 (0.34 to 1.64) | 2.12 (1.65 to 2.60) |
| Pakistan | 1.37 (0.56 to 2.31) | 1.79 (0.72 to 3.07) | 0.69 (0.46 to 0.92) |
| Palau | 2.06 (0.58 to 3.58) | 3.86 (1.01 to 7.25) | 2.36 (2.18 to 2.54) |
| Palestine | 1.01 (0.32 to 1.93) | 0.66 (0.22 to 1.24) | -1.68 (-1.80 to -1.57) |
| Panama | 0.71 (0.30 to 1.17) | 1.36 (0.51 to 2.46) | 2.36 (1.89 to 2.82) |
| Papua New Guinea | 0.59 (0.16 to 1.15) | 0.54 (0.13 to 1.04) | -0.38 (-0.48 to -0.28) |
| Paraguay | 1.55 (0.62 to 2.57) | 1.71 (0.67 to 3.20) | 0.52 (0.36 to 0.68) |
| Peru | 1.41 (0.43 to 2.58) | 1.78 (0.56 to 3.42) | 0.76 (0.45 to 1.06) |
| Philippines | 0.83 (0.22 to 1.53) | 1.17 (0.36 to 2.10) | 1.55 (1.43 to 1.68) |
| Poland | 0.47 (0.21 to 0.75) | 0.21 (0.10 to 0.35) | -3.03 (-3.77 to -2.28) |
| Portugal | 0.59 (0.26 to 0.97) | 0.51 (0.21 to 0.91) | -0.56 (-1.07 to -0.05) |
| Puerto Rico | 2.68 (1.08 to 4.59) | 2.64 (1.07 to 4.59) | 0.72 (0.17 to 1.28) |
| Qatar | 0.70 (0.25 to 1.40) | 1.11 (0.41 to 1.93) | 1.42 (0.74 to 2.11) |
| Republic of Korea | 0.99 (0.36 to 1.73) | 0.55 (0.19 to 0.96) | -2.37 (-2.68 to -2.06) |
| Republic of Moldova | 0.05 (0.02 to 0.08) | 0.09 (0.05 to 0.16) | 1.61 (1.06 to 2.17) |
| Romania | 0.15 (0.08 to 0.24) | 0.24 (0.12 to 0.37) | 2.71 (2.01 to 3.42) |
| Russian Federation | 0.26 (0.11 to 0.39) | 0.32 (0.15 to 0.49) | 0.24 (-0.27 to 0.76) |
| Rwanda | 1.09 (0.34 to 2.09) | 0.93 (0.31 to 1.82) | -1.31 (-1.66 to -0.96) |
| Saint Kitts and Nevis | 3.26 (1.35 to 5.47) | 4.16 (1.55 to 7.59) | 1.78 (1.45 to 2.12) |
| Saint Lucia | 2.39 (0.94 to 4.22) | 2.69 (1.03 to 4.83) | 0.91 (0.55 to 1.28) |
| Saint Vincent and the Grenadines | 1.52 (0.60 to 2.74) | 2.14 (0.85 to 3.82) | 1.72 (1.36 to 2.08) |
| Samoa | 1.85 (0.48 to 3.61) | 3.18 (0.91 to 5.79) | 1.96 (1.88 to 2.04) |
| San Marino | 0.37 (0.16 to 0.61) | 0.20 (0.08 to 0.36) | -0.61 (-1.05 to -0.17) |
| Sao Tome and Principe | 1.29 (0.39 to 2.35) | 1.29 (0.34 to 2.67) | -0.06 (-0.12 to 0.01) |
| Saudi Arabia | 1.24 (0.44 to 2.24) | 2.26 (0.74 to 4.01) | 1.56 (1.25 to 1.87) |
| Senegal | 1.21 (0.38 to 2.17) | 0.98 (0.29 to 1.86) | -0.98 (-1.15 to -0.80) |
| Serbia | 0.60 (0.24 to 1.08) | 0.42 (0.16 to 0.81) | -1.02 (-1.20 to -0.83) |
| Seychelles | 1.24 (0.35 to 2.38) | 1.56 (0.44 to 3.10) | 0.86 (0.69 to 1.04) |
| Sierra Leone | 0.84 (0.26 to 1.52) | 0.63 (0.20 to 1.25) | -1.00 (-1.09 to -0.91) |
| Singapore | 1.36 (0.61 to 2.25) | 0.81 (0.34 to 1.37) | -0.12 (-0.75 to 0.51) |
| Slovakia | 0.53 (0.25 to 0.83) | 0.40 (0.19 to 0.67) | -0.70 (-0.91 to -0.50) |
| Slovenia | 0.25 (0.14 to 0.38) | 0.23 (0.11 to 0.39) | 0.51 (0.14 to 0.88) |
| Solomon Islands | 1.76 (0.34 to 3.51) | 1.18 (0.27 to 2.16) | -1.58 (-1.82 to -1.34) |
| Somalia | 2.20 (0.72 to 4.26) | 2.60 (0.96 to 5.14) | 0.73 (0.57 to 0.89) |
| South Africa | 0.51 (0.19 to 0.93) | 0.66 (0.26 to 1.16) | 1.35 (1.06 to 1.65) |
| South Sudan | 1.85 (0.59 to 3.65) | 3.04 (1.09 to 5.81) | 1.55 (1.40 to 1.71) |
| Spain | 0.62 (0.28 to 1.03) | 0.35 (0.16 to 0.60) | -1.51 (-1.68 to -1.34) |
| Sri Lanka | 0.66 (0.17 to 1.37) | 0.60 (0.15 to 1.38) | -0.08 (-0.26 to 0.10) |
| Sudan | 0.37 (0.11 to 0.77) | 0.39 (0.12 to 0.78) | -0.06 (-0.38 to 0.25) |
| Suriname | 1.62 (0.59 to 2.83) | 2.01 (0.76 to 3.95) | 1.11 (0.89 to 1.33) |
| Sweden | 0.26 (0.12 to 0.41) | 0.53 (0.24 to 0.87) | 2.84 (2.66 to 3.02) |
| Switzerland | 0.25 (0.10 to 0.44) | 0.25 (0.09 to 0.43) | 1.05 (0.65 to 1.46) |
| Syrian Arab Republic | 0.99 (0.33 to 1.77) | 0.89 (0.31 to 1.74) | -0.88 (-1.21 to -0.55) |
| Taiwan (Province of China) | 2.18 (0.68 to 3.87) | 1.38 (0.42 to 2.47) | -1.14 (-1.51 to -0.78) |
| Tajikistan | 0.05 (0.02 to 0.09) | 0.06 (0.02 to 0.10) | -0.21 (-0.78 to 0.36) |
| Thailand | 0.70 (0.18 to 1.34) | 0.72 (0.22 to 1.37) | -0.21 (-0.34 to -0.09) |
| Timor-Leste | 0.87 (0.25 to 1.69) | 0.83 (0.22 to 1.95) | 0.03 (-0.32 to 0.38) |
| Togo | 0.95 (0.30 to 1.63) | 0.99 (0.31 to 1.90) | 0.01 (-0.12 to 0.13) |
| Tokelau | 1.38 (0.34 to 2.82) | 2.48 (0.72 to 4.80) | 2.18 (2.06 to 2.29) |
| Tonga | 0.81 (0.22 to 1.55) | 1.42 (0.38 to 2.76) | 1.71 (1.57 to 1.84) |
| Trinidad and Tobago | 1.85 (0.71 to 3.24) | 3.28 (1.30 to 6.04) | 2.51 (2.11 to 2.91) |
| Tunisia | 0.58 (0.23 to 1.08) | 0.61 (0.25 to 1.16) | 0.18 (0.11 to 0.26) |
| Turkmenistan | 0.38 (0.19 to 0.59) | 0.69 (0.31 to 1.16) | 1.48 (0.82 to 2.13) |
| Tuvalu | 1.80 (0.42 to 3.73) | 2.67 (0.68 to 4.95) | 1.44 (1.40 to 1.49) |
| Türkiye | 0.86 (0.31 to 1.57) | 0.58 (0.22 to 1.00) | -1.03 (-1.31 to -0.75) |
| Uganda | 1.41 (0.49 to 2.75) | 1.78 (0.60 to 3.46) | 0.40 (0.08 to 0.71) |
| Ukraine | 0.00 (0.00 to 0.01) | 0.05 (0.02 to 0.08) | 12.82 (10.80 to 14.87) |
| United Arab Emirates | 0.69 (0.24 to 1.26) | 1.23 (0.40 to 2.27) | 4.57 (3.75 to 5.39) |
| United Kingdom | 0.30 (0.15 to 0.46) | 0.28 (0.13 to 0.45) | 0.60 (0.32 to 0.88) |
| United Republic of Tanzania | 1.23 (0.53 to 2.07) | 1.41 (0.63 to 2.39) | 0.34 (0.28 to 0.39) |
| United States Virgin Islands | 1.12 (0.44 to 2.02) | 1.13 (0.42 to 2.08) | 0.44 (0.20 to 0.68) |
| United States of America | 0.77 (0.32 to 1.20) | 2.09 (0.78 to 3.20) | 3.34 (3.05 to 3.62) |
| Uruguay | 0.76 (0.29 to 1.23) | 0.71 (0.28 to 1.26) | 0.36 (0.00 to 0.72) |
| Uzbekistan | 0.22 (0.09 to 0.44) | 0.40 (0.18 to 0.65) | 0.57 (-0.32 to 1.48) |
| Vanuatu | 2.34 (0.78 to 4.86) | 2.65 (0.91 to 5.70) | 0.31 (0.21 to 0.41) |
| Venezuela (Bolivarian Republic of) | 0.86 (0.36 to 1.37) | 1.78 (0.66 to 3.26) | 2.00 (1.52 to 2.48) |
| Viet Nam | 0.67 (0.17 to 1.31) | 0.78 (0.21 to 1.62) | 1.06 (0.78 to 1.34) |
| Yemen | 0.70 (0.22 to 1.50) | 0.50 (0.16 to 0.95) | -1.42 (-1.63 to -1.21) |
| Zambia | 1.96 (0.66 to 3.65) | 2.42 (0.84 to 4.77) | 0.44 (0.28 to 0.60) |
| Zimbabwe | 0.57 (0.19 to 1.09) | 0.79 (0.27 to 1.61) | 1.36 (0.76 to 1.96) |

ASMR, Age-standardized mortality rate; T2D-related CKD, chronic kidney disease due to diabetes mellitus type 2; DALY, disability-adjusted life year; CI, confidence interval; EAPC, estimated annual percentage change

**Table S2 ASDR of T2D-related CKD attributable to dietary risks in 204 countries and territories, in 1990 and 2021, and the estimated annual percentage changes of DALY from 1990 to 2021**

| **location_name** | **ASDR per 100,000 people**  **(95% *CI*)** | | **EAPC of DALY, % (95% *CI*)** |
| --- | --- | --- | --- |
|  | **1990** | **2021** | **1990-2021** |
| Global | 20.55 (8.42 to 32.26) | 23.21 (9.95 to 36.61) | 0.47 (0.43 to 0.51) |
| East Asia | 21.51 (6.33 to 37.83) | 18.46 (5.75 to 32.68) | -0.45 (-0.59 to -0.31) |
| Southeast Asia | 19.28 (6.13 to 34.36) | 21.51 (6.85 to 39.70) | 0.47 (0.41 to 0.52) |
| Oceania | 28.15 (7.59 to 51.39) | 31.79 (9.99 to 57.16) | 0.33 (0.25 to 0.41) |
| Central Asia | 15.57 (7.85 to 23.69) | 16.80 (8.37 to 25.05) | -0.09 (-0.34 to 0.17) |
| Central Europe | 11.99 (6.10 to 18.00) | 9.62 (4.95 to 15.10) | -0.58 (-0.70 to -0.45) |
| Eastern Europe | 10.99 (5.08 to 16.43) | 9.00 (4.48 to 13.60) | -1.11 (-1.28 to -0.94) |
| High-income Asia Pacific | 29.20 (15.28 to 43.02) | 18.93 (8.98 to 28.32) | -1.37 (-1.51 to -1.23) |
| Australasia | 7.47 (3.44 to 11.52) | 8.37 (3.70 to 13.03) | 0.60 (0.35 to 0.84) |
| Western Europe | 14.04 (6.41 to 21.64) | 11.89 (5.35 to 18.39) | -0.37 (-0.47 to -0.26) |
| Southern Latin America | 32.57 (12.21 to 53.52) | 24.26 (9.93 to 39.12) | -0.61 (-0.92 to -0.30) |
| High-income North America | 23.00 (9.35 to 35.74) | 47.82 (17.94 to 73.13) | 2.53 (2.30 to 2.77) |
| Caribbean | 31.48 (12.93 to 50.63) | 39.96 (18.02 to 66.79) | 1.28 (1.11 to 1.45) |
| Andean Latin America | 33.58 (11.37 to 58.71) | 43.69 (16.77 to 76.52) | 0.95 (0.76 to 1.15) |
| Central Latin America | 28.83 (12.59 to 46.01) | 44.94 (20.14 to 71.41) | 1.97 (1.48 to 2.45) |
| Tropical Latin America | 34.39 (14.81 to 54.83) | 37.19 (16.04 to 59.24) | 0.15 (-0.09 to 0.38) |
| North Africa and Middle East | 20.37 (7.78 to 35.35) | 17.65 (6.78 to 29.90) | -0.48 (-0.55 to -0.41) |
| South Asia | 21.25 (8.66 to 34.94) | 25.33 (10.52 to 43.10) | 0.61 (0.53 to 0.68) |
| Central Sub-Saharan Africa | 38.50 (14.00 to 65.95) | 32.62 (11.15 to 58.23) | -0.80 (-0.89 to -0.70) |
| Eastern Sub-Saharan Africa | 39.33 (15.91 to 69.14) | 40.98 (17.42 to 69.45) | 0.00 (-0.05 to 0.05) |
| Southern Sub-Saharan Africa | 16.67 (6.45 to 29.41) | 19.57 (8.21 to 33.40) | 0.78 (0.54 to 1.02) |
| Western Sub-Saharan Africa | 20.69 (6.80 to 35.96) | 20.18 (7.41 to 33.81) | -0.10 (-0.16 to -0.04) |
| Afghanistan | 36.56 (11.99 to 70.67) | 32.13 (10.55 to 69.94) | -0.39 (-0.52 to -0.26) |
| Albania | 17.31 (8.89 to 25.60) | 12.07 (6.10 to 18.21) | -0.75 (-0.89 to -0.60) |
| Algeria | 18.51 (6.18 to 34.51) | 15.89 (5.90 to 28.97) | -0.68 (-0.89 to -0.47) |
| American Samoa | 52.16 (15.95 to 92.52) | 140.02 (43.91 to 257.50) | 3.46 (3.20 to 3.73) |
| Andorra | 19.31 (8.34 to 31.15) | 12.61 (5.61 to 20.00) | -1.12 (-1.26 to -0.99) |
| Angola | 25.37 (8.60 to 46.68) | 23.02 (8.25 to 42.88) | -0.59 (-0.70 to -0.47) |
| Antigua and Barbuda | 40.52 (15.84 to 68.28) | 57.51 (23.28 to 99.01) | 1.74 (1.46 to 2.01) |
| Argentina | 38.12 (13.25 to 64.79) | 27.30 (10.81 to 45.47) | -0.72 (-1.03 to -0.42) |
| Armenia | 7.34 (3.38 to 12.38) | 11.02 (5.00 to 18.01) | 1.19 (0.93 to 1.45) |
| Australia | 6.88 (3.15 to 10.73) | 7.70 (3.40 to 11.96) | 0.63 (0.43 to 0.83) |
| Austria | 13.14 (5.72 to 20.86) | 19.08 (8.82 to 29.22) | 2.01 (1.55 to 2.47) |
| Azerbaijan | 13.83 (6.85 to 21.79) | 12.97 (6.14 to 20.65) | -0.40 (-0.57 to -0.23) |
| Bahamas | 42.95 (17.71 to 70.62) | 53.81 (23.34 to 89.36) | 1.28 (1.07 to 1.49) |
| Bahrain | 16.97 (6.64 to 29.96) | 20.41 (7.64 to 36.10) | 0.19 (-0.04 to 0.41) |
| Bangladesh | 20.11 (7.35 to 35.26) | 19.22 (7.24 to 34.66) | 0.19 (0.07 to 0.31) |
| Barbados | 41.98 (18.06 to 68.58) | 52.53 (21.75 to 89.58) | 1.38 (1.07 to 1.68) |
| Belarus | 7.20 (3.08 to 11.78) | 7.50 (3.12 to 12.14) | -0.01 (-0.15 to 0.13) |
| Belgium | 15.91 (6.76 to 24.57) | 14.02 (6.40 to 21.92) | 0.24 (-0.07 to 0.56) |
| Belize | 33.26 (13.34 to 55.88) | 53.79 (21.80 to 89.99) | 1.94 (1.52 to 2.35) |
| Benin | 16.98 (4.73 to 30.81) | 15.85 (4.88 to 29.06) | -0.30 (-0.39 to -0.21) |
| Bermuda | 33.19 (12.60 to 54.98) | 30.17 (11.83 to 51.98) | 0.27 (-0.01 to 0.54) |
| Bhutan | 30.45 (11.39 to 53.17) | 28.33 (11.14 to 52.72) | -0.18 (-0.26 to -0.10) |
| Bolivia (Plurinational State of) | 53.47 (17.05 to 93.58) | 62.55 (21.28 to 114.75) | 0.72 (0.63 to 0.80) |
| Bosnia and Herzegovina | 15.62 (8.01 to 23.31) | 12.25 (6.06 to 19.77) | -0.82 (-1.14 to -0.51) |
| Botswana | 16.12 (5.71 to 28.93) | 16.29 (5.89 to 29.40) | 0.03 (-0.12 to 0.18) |
| Brazil | 34.32 (14.73 to 54.76) | 37.13 (16.02 to 59.39) | 0.14 (-0.10 to 0.39) |
| Brunei Darussalam | 67.88 (25.57 to 121.92) | 55.72 (20.93 to 100.50) | -0.32 (-0.49 to -0.15) |
| Bulgaria | 10.73 (5.42 to 16.36) | 16.00 (7.74 to 26.02) | 1.80 (1.54 to 2.06) |
| Burkina Faso | 20.00 (5.47 to 35.82) | 17.97 (5.71 to 31.31) | -0.37 (-0.46 to -0.27) |
| Burundi | 34.88 (12.45 to 65.34) | 32.21 (11.76 to 62.55) | -0.70 (-0.86 to -0.53) |
| Cabo Verde | 12.20 (4.14 to 21.46) | 14.24 (4.76 to 27.25) | 0.15 (-0.10 to 0.40) |
| Cambodia | 13.50 (3.01 to 25.46) | 11.22 (2.57 to 21.92) | -0.85 (-1.11 to -0.60) |
| Cameroon | 30.55 (8.40 to 56.81) | 34.05 (12.48 to 59.43) | 0.51 (0.19 to 0.82) |
| Canada | 9.37 (3.38 to 15.86) | 9.50 (3.38 to 16.06) | 0.35 (0.11 to 0.59) |
| Central African Republic | 46.16 (16.97 to 82.55) | 38.99 (14.33 to 71.68) | -0.59 (-0.66 to -0.52) |
| Chad | 21.03 (6.28 to 37.89) | 17.61 (4.82 to 31.68) | -0.76 (-0.91 to -0.61) |
| Chile | 20.39 (8.17 to 32.94) | 19.51 (8.15 to 31.36) | 0.17 (-0.25 to 0.60) |
| China | 21.06 (6.22 to 36.95) | 18.16 (5.68 to 32.15) | -0.45 (-0.59 to -0.31) |
| Colombia | 21.35 (8.62 to 35.32) | 18.95 (7.88 to 31.52) | 0.00 (-0.20 to 0.19) |
| Comoros | 30.27 (11.34 to 56.88) | 35.10 (13.19 to 64.97) | 0.34 (0.26 to 0.42) |
| Congo | 43.77 (15.11 to 78.08) | 37.54 (12.56 to 71.39) | -0.68 (-0.80 to -0.56) |
| Cook Islands | 27.72 (7.68 to 48.01) | 40.04 (12.87 to 72.67) | 1.32 (1.23 to 1.40) |
| Costa Rica | 26.97 (12.40 to 41.32) | 36.54 (15.26 to 58.23) | 1.08 (0.78 to 1.39) |
| Croatia | 10.20 (4.81 to 15.91) | 9.97 (4.59 to 16.38) | -0.15 (-0.34 to 0.05) |
| Cuba | 18.53 (7.94 to 29.81) | 25.61 (11.18 to 42.20) | 1.58 (1.32 to 1.85) |
| Cyprus | 22.90 (10.00 to 36.55) | 13.93 (6.52 to 21.85) | -1.66 (-1.87 to -1.45) |
| Czechia | 11.55 (5.64 to 17.56) | 6.95 (3.09 to 11.23) | -1.59 (-1.72 to -1.46) |
| Côte d'Ivoire | 21.00 (6.72 to 36.74) | 17.48 (6.18 to 30.35) | -0.73 (-0.81 to -0.64) |
| Democratic People's Republic of Korea | 21.70 (6.04 to 39.04) | 19.03 (5.11 to 34.97) | -0.37 (-0.51 to -0.24) |
| Democratic Republic of the Congo | 40.70 (14.70 to 72.06) | 34.29 (11.74 to 61.93) | -0.82 (-0.93 to -0.71) |
| Denmark | 12.65 (5.84 to 19.01) | 16.90 (8.08 to 25.58) | 0.85 (0.71 to 0.99) |
| Djibouti | 33.53 (12.36 to 62.10) | 53.41 (20.86 to 95.86) | 1.51 (1.33 to 1.68) |
| Dominica | 37.25 (15.36 to 60.88) | 53.46 (22.74 to 97.71) | 1.43 (1.34 to 1.53) |
| Dominican Republic | 23.92 (8.93 to 41.59) | 30.62 (11.05 to 54.96) | 1.48 (1.26 to 1.70) |
| Ecuador | 30.66 (11.09 to 51.95) | 47.16 (17.89 to 87.90) | 1.43 (0.60 to 2.27) |
| Egypt | 26.24 (7.65 to 47.14) | 22.79 (8.73 to 40.62) | -0.44 (-0.53 to -0.36) |
| El Salvador | 30.40 (11.91 to 52.21) | 66.56 (27.62 to 119.65) | 2.62 (2.13 to 3.12) |
| Equatorial Guinea | 39.84 (13.26 to 69.81) | 42.62 (15.65 to 81.59) | 0.44 (0.05 to 0.83) |
| Eritrea | 27.39 (9.11 to 57.54) | 32.98 (11.04 to 68.72) | 0.64 (0.57 to 0.71) |
| Estonia | 13.02 (5.85 to 20.52) | 21.14 (9.27 to 31.59) | 1.38 (1.17 to 1.59) |
| Eswatini | 24.59 (7.83 to 42.73) | 29.72 (10.96 to 57.34) | 0.78 (0.26 to 1.30) |
| Ethiopia | 62.14 (16.22 to 116.12) | 44.61 (15.68 to 81.66) | -1.42 (-1.55 to -1.30) |
| Fiji | 53.98 (15.37 to 99.10) | 100.78 (33.06 to 187.59) | 1.65 (1.34 to 1.97) |
| Finland | 10.55 (5.61 to 15.29) | 9.17 (4.84 to 13.71) | 0.05 (-0.20 to 0.30) |
| France | 12.77 (5.35 to 19.43) | 11.40 (4.84 to 17.44) | -0.36 (-0.52 to -0.20) |
| Gabon | 41.32 (13.89 to 76.67) | 47.18 (15.69 to 83.02) | 0.32 (0.14 to 0.50) |
| Gambia | 22.30 (6.71 to 39.44) | 22.57 (7.04 to 43.72) | -0.14 (-0.28 to 0.01) |
| Georgia | 12.59 (6.45 to 19.24) | 19.39 (10.45 to 29.45) | 1.65 (1.27 to 2.03) |
| Germany | 16.21 (7.85 to 24.75) | 14.44 (6.68 to 22.29) | -0.20 (-0.44 to 0.04) |
| Ghana | 21.33 (7.19 to 37.91) | 30.34 (11.49 to 52.14) | 1.56 (1.31 to 1.81) |
| Greece | 22.18 (8.61 to 36.97) | 21.10 (9.07 to 34.37) | -0.39 (-0.94 to 0.17) |
| Greenland | 20.10 (6.94 to 34.79) | 18.04 (6.74 to 30.60) | 0.04 (-0.09 to 0.16) |
| Grenada | 47.78 (18.42 to 86.14) | 74.92 (29.09 to 133.94) | 1.99 (1.80 to 2.19) |
| Guam | 36.90 (11.92 to 63.68) | 38.74 (12.56 to 68.26) | 0.93 (0.61 to 1.25) |
| Guatemala | 28.91 (10.37 to 48.67) | 42.83 (16.73 to 78.71) | 2.33 (1.74 to 2.94) |
| Guinea | 14.12 (3.81 to 25.17) | 12.13 (3.66 to 22.61) | -0.32 (-0.39 to -0.25) |
| Guinea-Bissau | 28.95 (8.95 to 51.70) | 21.76 (6.94 to 38.64) | -1.01 (-1.05 to -0.96) |
| Guyana | 44.98 (17.07 to 78.40) | 82.49 (30.49 to 155.16) | 3.05 (2.67 to 3.42) |
| Haiti | 45.48 (17.40 to 87.82) | 52.31 (17.86 to 115.06) | 0.76 (0.66 to 0.86) |
| Honduras | 16.29 (6.75 to 28.11) | 24.68 (9.62 to 44.52) | 1.59 (1.42 to 1.76) |
| Hungary | 9.43 (4.85 to 14.06) | 9.03 (4.31 to 14.05) | 0.47 (0.10 to 0.85) |
| Iceland | 7.97 (3.40 to 12.75) | 7.18 (3.02 to 11.45) | -0.30 (-0.42 to -0.19) |
| India | 19.63 (8.13 to 32.93) | 24.03 (9.87 to 41.77) | 0.72 (0.64 to 0.81) |
| Indonesia | 20.15 (6.17 to 38.10) | 22.91 (6.87 to 47.27) | 0.47 (0.42 to 0.51) |
| Iran (Islamic Republic of) | 13.47 (4.20 to 24.30) | 8.54 (2.92 to 15.22) | -1.54 (-1.70 to -1.38) |
| Iraq | 29.54 (11.52 to 50.51) | 25.54 (8.96 to 50.43) | -0.68 (-0.85 to -0.51) |
| Ireland | 18.59 (9.08 to 28.63) | 14.69 (6.96 to 22.36) | -0.58 (-0.69 to -0.47) |
| Israel | 18.82 (7.78 to 30.86) | 12.18 (5.08 to 19.85) | -1.04 (-1.41 to -0.66) |
| Italy | 12.14 (5.20 to 18.94) | 8.69 (3.59 to 13.69) | -1.08 (-1.12 to -1.03) |
| Jamaica | 24.58 (8.85 to 42.51) | 37.73 (15.00 to 70.49) | 1.10 (0.50 to 1.71) |
| Japan | 30.75 (16.31 to 44.81) | 21.64 (10.39 to 32.27) | -1.10 (-1.23 to -0.97) |
| Jordan | 29.75 (11.13 to 50.30) | 30.53 (11.31 to 51.88) | -0.09 (-0.50 to 0.31) |
| Kazakhstan | 19.45 (10.47 to 29.46) | 18.92 (9.96 to 28.19) | -0.42 (-0.62 to -0.21) |
| Kenya | 28.76 (11.83 to 52.23) | 44.65 (18.70 to 76.94) | 1.73 (1.61 to 1.85) |
| Kiribati | 45.89 (11.04 to 82.47) | 58.86 (12.79 to 117.48) | 0.79 (0.66 to 0.92) |
| Kuwait | 17.49 (6.44 to 30.43) | 10.18 (3.48 to 18.22) | -1.48 (-1.72 to -1.24) |
| Kyrgyzstan | 15.37 (6.91 to 24.78) | 16.52 (7.45 to 26.25) | -0.51 (-0.94 to -0.07) |
| Lao People's Democratic Republic | 35.98 (8.97 to 69.57) | 23.49 (6.23 to 48.79) | -1.58 (-1.68 to -1.47) |
| Latvia | 12.06 (5.31 to 19.12) | 15.68 (7.28 to 23.69) | 0.98 (0.85 to 1.11) |
| Lebanon | 13.18 (4.44 to 23.61) | 11.52 (4.04 to 20.62) | -0.26 (-0.51 to -0.02) |
| Lesotho | 11.63 (3.43 to 21.15) | 18.53 (6.50 to 33.81) | 2.01 (1.67 to 2.35) |
| Liberia | 25.05 (7.86 to 43.26) | 21.39 (6.84 to 39.11) | -0.28 (-0.58 to 0.02) |
| Libya | 15.63 (5.94 to 26.74) | 21.70 (8.00 to 41.00) | 1.59 (1.43 to 1.74) |
| Lithuania | 11.83 (5.32 to 18.53) | 14.83 (6.84 to 22.64) | 0.50 (0.35 to 0.66) |
| Luxembourg | 21.79 (9.60 to 32.60) | 13.32 (6.26 to 20.69) | -1.59 (-1.85 to -1.33) |
| Madagascar | 27.40 (9.32 to 50.24) | 32.09 (10.86 to 59.03) | 0.59 (0.52 to 0.66) |
| Malawi | 41.40 (15.29 to 73.45) | 52.89 (21.48 to 94.27) | 0.61 (0.34 to 0.87) |
| Malaysia | 34.61 (9.95 to 63.52) | 36.43 (11.09 to 66.34) | 0.26 (-0.01 to 0.53) |
| Maldives | 41.48 (12.28 to 78.64) | 17.96 (4.66 to 36.52) | -3.10 (-3.31 to -2.89) |
| Mali | 25.19 (7.49 to 45.50) | 20.32 (6.42 to 35.76) | -0.57 (-0.66 to -0.48) |
| Malta | 21.28 (9.51 to 32.18) | 15.02 (6.60 to 23.28) | -1.09 (-1.42 to -0.77) |
| Marshall Islands | 41.68 (8.78 to 91.12) | 83.77 (10.69 to 238.84) | 2.49 (2.34 to 2.64) |
| Mauritania | 32.97 (9.87 to 59.89) | 30.31 (10.20 to 52.59) | -0.55 (-0.66 to -0.43) |
| Mauritius | 40.77 (10.82 to 76.06) | 88.09 (29.21 to 150.50) | 2.87 (2.42 to 3.33) |
| Mexico | 33.92 (15.04 to 55.33) | 56.89 (26.64 to 89.09) | 2.42 (1.73 to 3.11) |
| Micronesia (Federated States of) | 58.28 (12.94 to 111.29) | 82.46 (19.40 to 162.12) | 1.35 (1.20 to 1.50) |
| Monaco | 14.68 (6.59 to 21.99) | 15.40 (6.38 to 23.93) | 0.28 (0.14 to 0.42) |
| Mongolia | 39.19 (20.42 to 57.78) | 31.04 (16.71 to 44.94) | -1.08 (-1.20 to -0.96) |
| Montenegro | 11.09 (4.54 to 18.29) | 9.22 (3.47 to 16.07) | -0.54 (-0.65 to -0.43) |
| Morocco | 18.22 (7.49 to 32.29) | 16.21 (7.29 to 27.46) | -0.23 (-0.51 to 0.04) |
| Mozambique | 29.74 (10.65 to 55.88) | 43.38 (16.56 to 78.01) | 1.60 (1.42 to 1.79) |
| Myanmar | 23.65 (5.54 to 47.67) | 18.72 (5.20 to 36.91) | -0.87 (-0.92 to -0.82) |
| Namibia | 18.26 (6.38 to 32.77) | 16.24 (5.31 to 29.58) | -0.67 (-0.94 to -0.41) |
| Nauru | 57.51 (16.51 to 111.92) | 97.22 (27.87 to 196.07) | 1.62 (1.50 to 1.73) |
| Nepal | 25.64 (9.86 to 43.91) | 28.07 (10.28 to 48.90) | 0.37 (0.23 to 0.51) |
| Netherlands | 9.77 (3.88 to 15.58) | 11.14 (4.86 to 17.50) | 0.25 (-0.01 to 0.52) |
| New Zealand | 10.49 (4.59 to 16.43) | 11.80 (5.04 to 18.47) | 0.49 (0.01 to 0.98) |
| Nicaragua | 33.43 (13.04 to 59.10) | 62.22 (23.09 to 111.34) | 2.56 (2.14 to 2.98) |
| Niger | 18.21 (5.58 to 31.82) | 12.87 (4.54 to 24.32) | -1.16 (-1.21 to -1.10) |
| Nigeria | 19.00 (6.08 to 33.69) | 18.06 (6.63 to 30.40) | -0.33 (-0.49 to -0.16) |
| Niue | 51.31 (16.28 to 96.05) | 102.23 (28.29 to 222.74) | 2.27 (2.19 to 2.35) |
| North Macedonia | 10.64 (4.98 to 16.86) | 9.47 (4.25 to 15.78) | -0.24 (-0.58 to 0.10) |
| Northern Mariana Islands | 79.82 (24.24 to 136.38) | 90.47 (30.94 to 161.47) | 0.56 (0.29 to 0.84) |
| Norway | 11.36 (5.27 to 17.33) | 11.59 (5.19 to 17.59) | 0.06 (-0.16 to 0.28) |
| Oman | 15.40 (4.64 to 28.94) | 20.60 (7.78 to 37.53) | 1.80 (1.47 to 2.12) |
| Pakistan | 33.94 (13.87 to 55.23) | 44.56 (19.01 to 73.90) | 0.70 (0.51 to 0.89) |
| Palau | 48.33 (13.18 to 83.77) | 80.28 (21.98 to 149.44) | 1.91 (1.77 to 2.05) |
| Palestine | 22.69 (7.34 to 41.93) | 14.67 (5.01 to 26.41) | -1.73 (-1.83 to -1.63) |
| Panama | 21.18 (9.41 to 33.63) | 33.72 (13.87 to 58.54) | 1.73 (1.41 to 2.06) |
| Papua New Guinea | 16.90 (4.45 to 32.24) | 14.56 (3.71 to 27.96) | -0.57 (-0.68 to -0.46) |
| Paraguay | 36.98 (14.70 to 60.02) | 39.63 (16.51 to 70.96) | 0.27 (0.12 to 0.42) |
| Peru | 29.62 (8.97 to 53.91) | 37.18 (11.91 to 71.04) | 0.75 (0.46 to 1.04) |
| Philippines | 18.37 (4.80 to 33.45) | 28.69 (9.15 to 51.91) | 1.83 (1.70 to 1.97) |
| Poland | 14.14 (6.77 to 21.75) | 7.67 (3.52 to 11.95) | -2.17 (-2.60 to -1.75) |
| Portugal | 14.20 (6.23 to 22.21) | 10.98 (4.63 to 17.70) | -0.74 (-1.17 to -0.30) |
| Puerto Rico | 57.63 (24.04 to 92.54) | 56.78 (23.95 to 95.07) | 0.66 (0.18 to 1.14) |
| Qatar | 16.19 (6.19 to 29.90) | 22.18 (8.22 to 38.61) | 0.99 (0.46 to 1.53) |
| Republic of Korea | 18.84 (7.07 to 32.69) | 10.30 (3.74 to 17.80) | -2.25 (-2.56 to -1.94) |
| Republic of Moldova | 8.47 (3.56 to 14.01) | 9.50 (4.32 to 15.23) | 0.21 (0.08 to 0.34) |
| Romania | 8.91 (4.82 to 13.37) | 10.99 (5.96 to 16.32) | 1.32 (0.96 to 1.68) |
| Russian Federation | 13.61 (6.22 to 19.93) | 9.80 (4.92 to 14.78) | -1.66 (-1.88 to -1.44) |
| Rwanda | 23.75 (7.82 to 46.56) | 18.76 (6.11 to 38.69) | -1.63 (-1.99 to -1.26) |
| Saint Kitts and Nevis | 77.30 (33.77 to 125.52) | 92.25 (36.08 to 165.08) | 1.45 (1.13 to 1.78) |
| Saint Lucia | 53.42 (21.89 to 89.75) | 59.60 (23.30 to 102.84) | 0.94 (0.62 to 1.26) |
| Saint Vincent and the Grenadines | 36.17 (14.82 to 61.98) | 49.85 (20.62 to 84.24) | 1.57 (1.25 to 1.89) |
| Samoa | 45.09 (11.61 to 89.55) | 71.71 (20.26 to 132.92) | 1.70 (1.61 to 1.79) |
| San Marino | 12.29 (5.41 to 19.08) | 8.93 (3.84 to 14.07) | -0.53 (-0.70 to -0.37) |
| Sao Tome and Principe | 28.04 (8.93 to 52.12) | 29.90 (8.50 to 59.53) | 0.09 (-0.01 to 0.20) |
| Saudi Arabia | 31.13 (11.44 to 55.20) | 54.24 (17.80 to 92.18) | 1.42 (1.11 to 1.74) |
| Senegal | 29.25 (9.39 to 51.69) | 22.71 (7.41 to 42.16) | -1.08 (-1.24 to -0.92) |
| Serbia | 14.82 (6.75 to 24.65) | 10.43 (4.20 to 18.72) | -1.16 (-1.29 to -1.02) |
| Seychelles | 33.68 (9.26 to 61.61) | 36.45 (10.24 to 68.93) | 0.33 (0.18 to 0.49) |
| Sierra Leone | 20.41 (6.55 to 36.04) | 16.57 (5.61 to 30.77) | -0.73 (-0.80 to -0.65) |
| Singapore | 29.40 (13.37 to 48.31) | 16.69 (6.93 to 27.53) | -0.43 (-0.94 to 0.09) |
| Slovakia | 16.60 (8.47 to 25.08) | 12.36 (6.44 to 19.56) | -0.85 (-0.97 to -0.72) |
| Slovenia | 10.74 (5.78 to 15.51) | 8.71 (4.54 to 13.19) | -0.55 (-0.75 to -0.35) |
| Solomon Islands | 42.92 (8.02 to 87.62) | 29.43 (6.54 to 53.09) | -1.46 (-1.68 to -1.24) |
| Somalia | 48.23 (16.20 to 92.77) | 56.51 (20.27 to 111.63) | 0.65 (0.49 to 0.80) |
| South Africa | 16.57 (6.48 to 28.35) | 19.21 (8.36 to 32.57) | 0.79 (0.58 to 0.99) |
| South Sudan | 39.50 (12.80 to 74.79) | 63.44 (24.95 to 118.49) | 1.47 (1.28 to 1.66) |
| Spain | 16.08 (6.84 to 26.10) | 9.22 (4.10 to 14.85) | -1.47 (-1.68 to -1.26) |
| Sri Lanka | 15.58 (3.96 to 31.29) | 15.15 (4.04 to 31.65) | 0.10 (-0.09 to 0.29) |
| Sudan | 10.86 (3.24 to 20.48) | 10.53 (3.57 to 20.48) | -0.30 (-0.54 to -0.07) |
| Suriname | 41.28 (15.29 to 70.58) | 49.82 (18.48 to 93.58) | 0.89 (0.68 to 1.10) |
| Sweden | 9.88 (4.61 to 15.34) | 12.02 (5.30 to 18.78) | 1.14 (1.00 to 1.28) |
| Switzerland | 9.70 (3.63 to 16.06) | 7.93 (2.94 to 13.10) | -0.19 (-0.45 to 0.07) |
| Syrian Arab Republic | 23.66 (8.40 to 41.57) | 21.16 (7.67 to 39.59) | -0.88 (-1.20 to -0.57) |
| Taiwan (Province of China) | 42.40 (12.66 to 75.16) | 30.33 (8.95 to 53.72) | -0.50 (-0.78 to -0.21) |
| Tajikistan | 7.99 (3.52 to 13.15) | 7.05 (3.29 to 11.41) | -0.71 (-0.89 to -0.53) |
| Thailand | 17.20 (4.35 to 33.18) | 18.16 (5.51 to 36.02) | 0.00 (-0.10 to 0.10) |
| Timor-Leste | 19.99 (5.53 to 39.62) | 19.92 (5.24 to 44.99) | 0.12 (-0.17 to 0.40) |
| Togo | 22.47 (7.47 to 38.94) | 23.33 (7.72 to 41.17) | 0.00 (-0.13 to 0.12) |
| Tokelau | 32.96 (8.19 to 67.39) | 52.56 (15.60 to 101.21) | 1.75 (1.66 to 1.84) |
| Tonga | 22.99 (6.51 to 42.86) | 34.45 (9.37 to 67.08) | 1.13 (1.03 to 1.24) |
| Trinidad and Tobago | 42.42 (17.44 to 70.85) | 77.86 (32.66 to 141.20) | 2.51 (2.13 to 2.89) |
| Tunisia | 14.55 (5.96 to 25.47) | 13.98 (5.64 to 25.37) | -0.20 (-0.28 to -0.13) |
| Turkmenistan | 20.72 (10.75 to 31.62) | 26.32 (13.13 to 41.69) | 0.65 (0.29 to 1.02) |
| Tuvalu | 44.74 (10.33 to 87.51) | 60.95 (15.58 to 115.20) | 1.12 (1.07 to 1.16) |
| Türkiye | 20.34 (7.30 to 35.69) | 13.37 (4.95 to 22.98) | -1.04 (-1.28 to -0.80) |
| Uganda | 29.62 (10.66 to 57.06) | 36.09 (13.24 to 69.48) | 0.25 (-0.07 to 0.57) |
| Ukraine | 5.06 (2.17 to 8.29) | 5.68 (2.43 to 9.17) | 0.50 (0.34 to 0.65) |
| United Arab Emirates | 19.14 (6.88 to 33.48) | 25.62 (9.03 to 46.84) | 2.89 (2.30 to 3.49) |
| United Kingdom | 13.57 (6.68 to 20.56) | 11.45 (5.22 to 17.77) | -0.28 (-0.38 to -0.18) |
| United Republic of Tanzania | 28.08 (12.39 to 46.49) | 30.83 (14.70 to 49.88) | 0.24 (0.20 to 0.28) |
| United States Virgin Islands | 27.96 (11.18 to 47.60) | 27.32 (10.75 to 49.60) | 0.32 (0.13 to 0.52) |
| United States of America | 24.46 (10.01 to 37.89) | 52.47 (19.70 to 80.30) | 2.63 (2.39 to 2.88) |
| Uruguay | 17.72 (6.63 to 28.65) | 16.20 (6.34 to 26.91) | 0.19 (-0.11 to 0.49) |
| Uzbekistan | 14.31 (6.57 to 22.89) | 16.47 (7.76 to 25.19) | -0.21 (-0.69 to 0.27) |
| Vanuatu | 54.02 (17.97 to 110.54) | 61.37 (21.80 to 127.94) | 0.33 (0.26 to 0.41) |
| Venezuela (Bolivarian Republic of) | 25.20 (11.19 to 38.63) | 43.04 (17.41 to 74.37) | 1.33 (0.93 to 1.73) |
| Viet Nam | 14.64 (3.62 to 28.59) | 16.34 (4.51 to 31.82) | 0.92 (0.65 to 1.19) |
| Yemen | 18.04 (5.80 to 35.71) | 13.02 (4.29 to 23.94) | -1.28 (-1.45 to -1.11) |
| Zambia | 43.13 (14.91 to 79.50) | 54.42 (20.00 to 105.86) | 0.51 (0.35 to 0.66) |
| Zimbabwe | 17.24 (5.86 to 31.46) | 22.53 (8.18 to 42.08) | 1.01 (0.50 to 1.53) |

ASDR, Age-standardized DALY rate; T2D-related CKD, chronic kidney disease due to diabetes mellitus type 2; DALY, disability-adjusted life year; CI, confidence interval; EAPC, estimated annual percentage change

**Figure legend**

**Fig. S1 The global burden of chronic kidney disease deaths and DALYs due to diabetes mellitus type 2 attributable to dietary risks in different regions in 2021.**


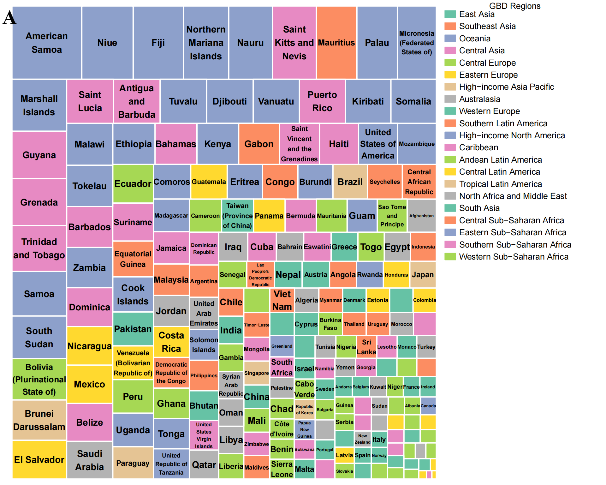

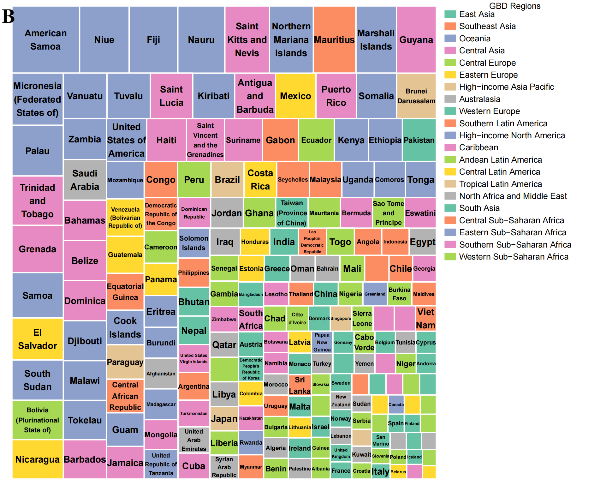


Deaths (A) and DALYs (B) of T2D-related CKD attributable to dietary risks.

DALYs, disability-adjusted life years; T2D-related CKD, chronic kidney disease due to type 2

**Fig. S2 The detailed dietary risks for chronic kidney disease deaths and DALYs due to diabetes mellitus type 2 by sex from1990 to 2021.**


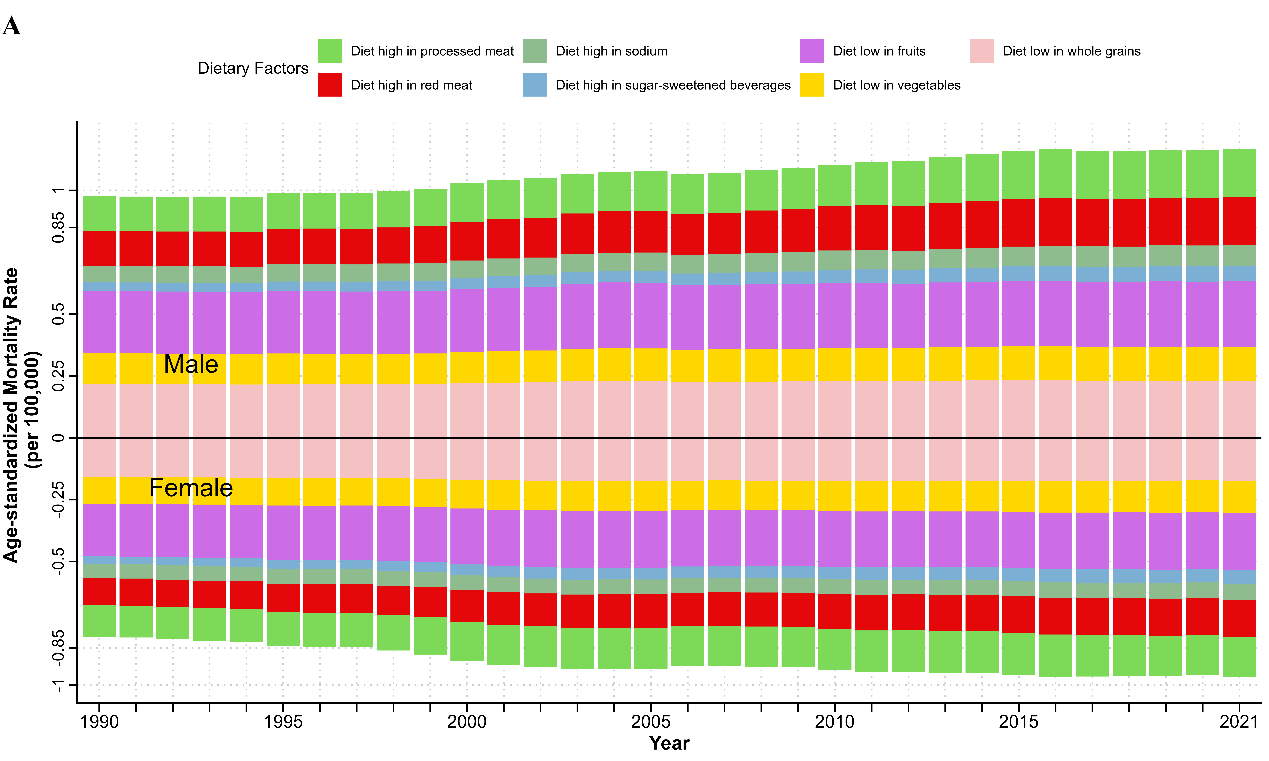

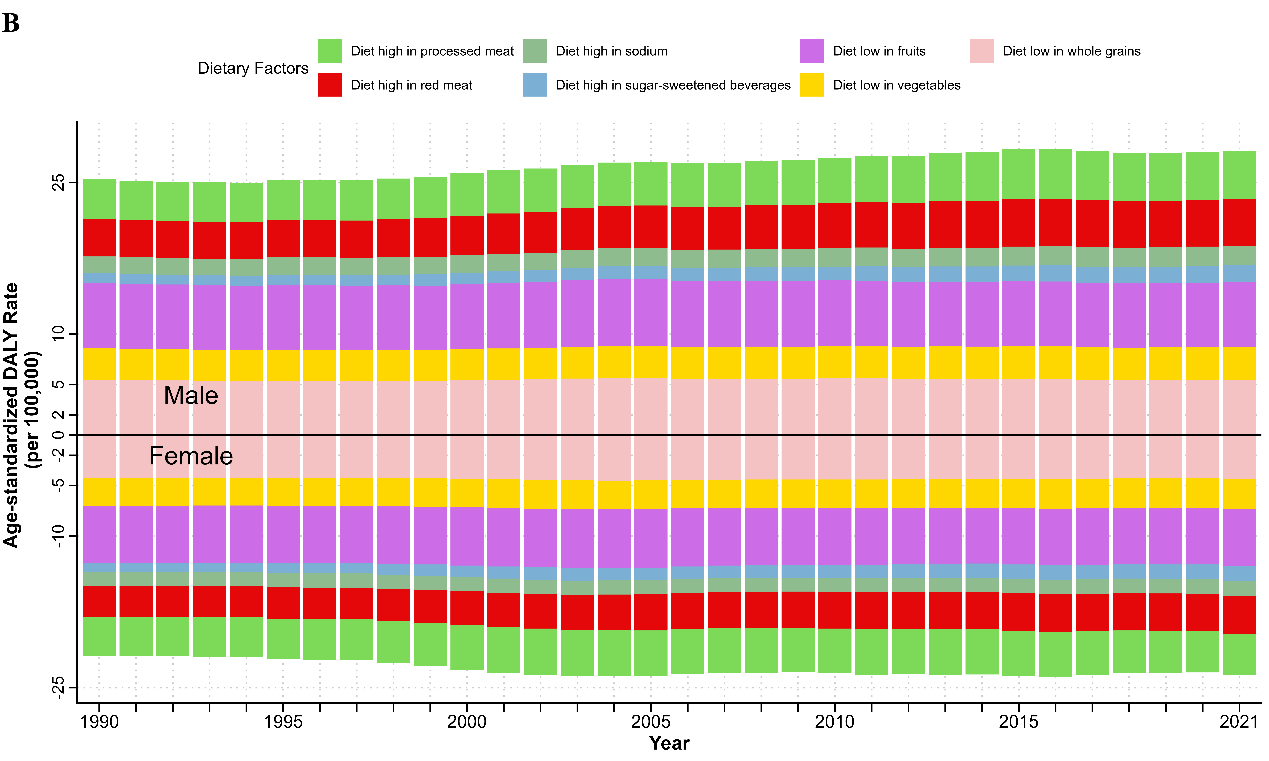


ASMR (A) and ASDR (B) of T2D-related CKD attributable to dietary risks.

ASMR, age-standardized mortality rate; ASDR, age-standardized DALYs rate; DALYs, disability-adjusted life years; T2D-related CKD, chronic kidney disease due to type 2 diabetes.

**Fig. S3 The trends for chronic kidney disease deaths and DALYs due to diabetes mellitus type 2 attributable to dietary risks in <70 years, 70-74 years, 75-79 years and ≥80 years by sex from 1990 to 2021.**


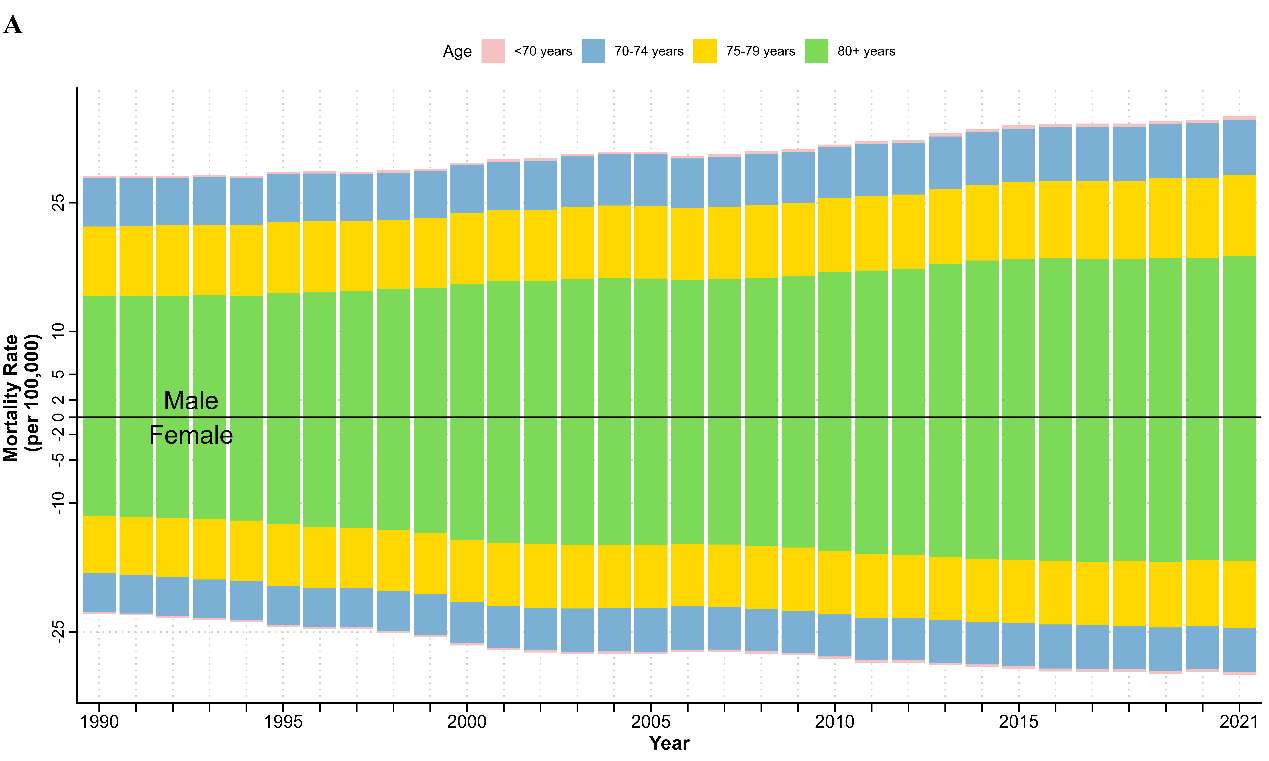


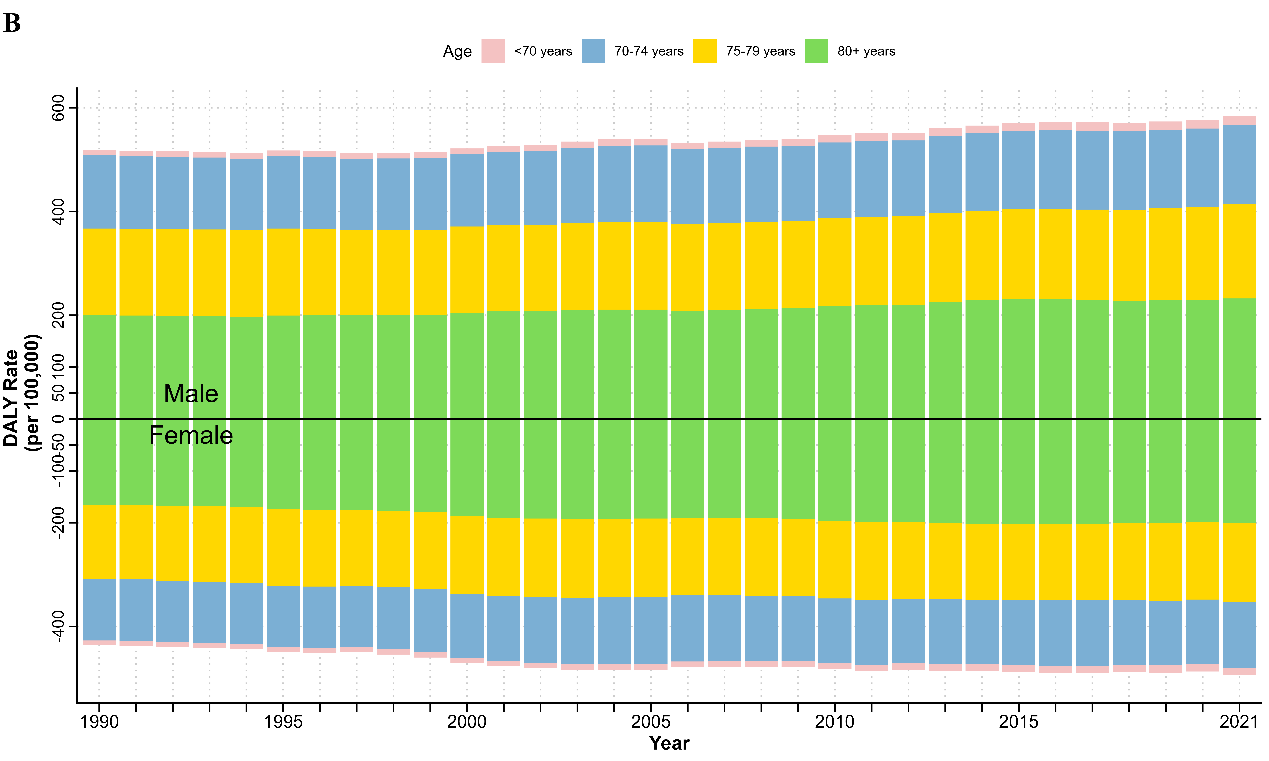


Mortality Rate per 100,000 people and DALY Rate (B) of T2D-related CKD attributable to dietary risks.

DALYs, disability-adjusted life years; T2D-related CKD, chronic kidney disease due to type 2 diabetes
